# Extensions to Multivariate Space Time Mixture Modeling of Small Area Cancer Data

**DOI:** 10.3390/ijerph14050503

**Published:** 2017-05-09

**Authors:** Rachel Carroll, Andrew B. Lawson, Christel Faes, Russell S. Kirby, Mehreteab Aregay, Kevin Watjou

**Affiliations:** 1 Department of Public Health Sciences, Medical University of South Carolina, 135 Cannon St, Charleston, SC 29425, USA; lawsonab@musc.edu (A.B.L.); mhrete_ab@yahoo.com (M.A.); 2 Interuniversity Institute for Statistics and Statistical Bioinformatics, Hasselt University, 3500 Hasselt, Belgium; christel.faes@uhasselt.be (C.F.); kevin.watjou@uhasselt.be (K.W.); 3 Department of Community and Family Health, University of South Florida, Tampa, FL 33620, USA; rkirby@health.usf.edu

**Keywords:** lung and bronchus cancer, melanoma cancer of the skin, oral cavity and pharynx cancer, incidence, mixture model, spatio-temporal, disease mapping

## Abstract

Oral cavity and pharynx cancer, even when considered together, is a fairly rare disease. Implementation of multivariate modeling with lung and bronchus cancer, as well as melanoma cancer of the skin, could lead to better inference for oral cavity and pharynx cancer. The multivariate structure of these models is accomplished via the use of shared random effects, as well as other multivariate prior distributions. The results in this paper indicate that care should be taken when executing these types of models, and that multivariate mixture models may not always be the ideal option, depending on the data of interest.

## 1. Introduction

Many cancers share risk factors but differ in incidence. Previous studies have demonstrated benefits for rarer diseases via the implementation of multivariate modeling with a more prevalent disease that presents common risk factors [1,2]. Oral cavity and pharynx cancer (hereafter referred to as oral/pharynx) (ICD-9-CM codes: 145.9, 146.2, 146.3, 146.5–146.9, 147.0–147.3, 147.8, 147.9, 148.9, 149.0, 149.8, 149.9) and lung and bronchus cancer (henceforth referred to as lung) (ICD-9-CM codes: 162.2–162.5, 162.8, and 162.9) share tobacco smoke as an exposure, while lip cancer, which is included with oral/pharynx cancer, and melanoma cancer of the skin (referred to as melanoma from here) (ICD-9-CM codes: 172.2, 172.3, 172.5–172.7, and 172.9), have the common risk factor of ultraviolet light exposure [3,4,5,6]. Additionally, oral/pharynx is quite rare in comparison to lung and melanoma, which are among the most common types of cancer in the United States [7]. Thus, it is of interest to explore the benefits of employing multivariate modeling for these relationships by including all three diseases in a single model.

Oral/pharynx, melanoma, and lung all display spatio-temporal (ST) patterns in their incidences [8,9,10,11,12], and a Bayesian multivariate ST mixture model proposed by Lawson et al. [2] can offer an ideal, flexible model for risk that offers improvements over a more standard multivariate model introduced by Knorr-Held [13,14,15]. The real data example in that paper also involved the three diseases of interest and illustrated that care should be taken in implementing types of models. In that example, there was evidence that modeling was improved for oral/pharynx, but only up to a certain point in time, year 10, in the multivariate setting. To deal with this, they simply restricted to the first 10 years of the study time to create a working example. However, in this paper we wish to explore extensions to the model that could offer greater temporal flexibility for situations of this nature so that all of the data can be utilized and no information is lost. These extensions will involve imposing more flexibility in the mixture model, as well as bivariate fits of only oral/pharynx with lung because that relationship is more grounded in the literature.

Often, some important risk factors may be unavailable because they are difficult to measure or obtain. A benefit of employing an ST model involves the models’ ability to account for such unmeasured exposures that may be common among the diseases considered. Examples of these exposures include: health service availability, environmental influences, or demographic factors. In this study, we do not have the ability to measure many of these exposures since data are aggregated at the county level.

This paper is developed as follows: First, we describe the case study and the statistical methods. Following that, we present the results. Finally, we discuss the findings and draw conclusions.

## 2. Materials and Methods

The outcome data of interest is in the form of the annual South Carolina county level incidences for oral/pharynx, lung, and melanoma over the years 1996–2009. A conditionally-independent Poisson distribution is a commonly-used model for small area counts in disease mapping [16,17,18], and is described as follows:

$$y_{ijk}\sim Pois\left(\mu_{ijk}\right)$$

$$\mu_{ijk}=e_{ijk}\theta_{ijk}$$

with $y_{ijk}$, the incidence of cancer k in county i at time j, and $\mu_{ijk}$, the mean of the Poisson distribution defined as the product of the known expected rate of disease, $e_{ijk}$, and the relative risk, $\theta_{ijk}$. In this framework, we model the logarithm of the relative risk; these modeling options are described in Section 3.

### 2.1. Case Study

The outcome data for this analysis was gathered from the South Carolina Assessment Network [19]. In this data, the managers placed thresholds on small counts of disease such that an observed count between 1 and 4 for a particular county is given the value 5 and an observed count between 5 and 9 is given the value 10. To make the distribution of counts more appropriate, we imputed values based on the appropriate interval as indicated by the threshold value. To do this, we calculate an expected count of diseases for each county and year by multiplying the overall rate of disease (based on the threshold values) by the county population. Next, we sample from a Poisson distribution with the expected rate as the mean restricted such that the imputed value falls within the appropriate threshold. The code for accomplishing this is included in the supplemental files of Lawson et al. [2]. The numbers and percentages of zeros and imputed data for oral/pharynx, melanoma, and lung are displayed in Table 1.

**Figure 1:**
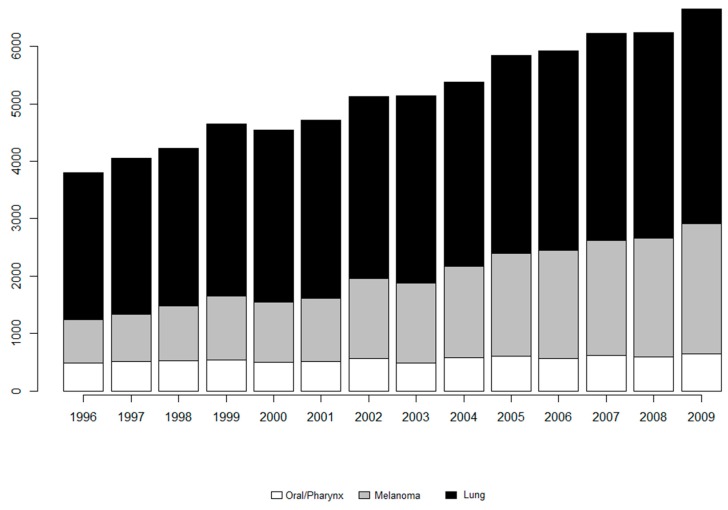
Bar plot of incidences of all cancers over time.

Following the imputation, the expected rate of disease ($e_{ijk}$) is calculated. The subsequent statistics and figures are based on the imputed dataset. Figure 1 displays a histogram of incidences per study year segmented by disease. From this, it is easy to see that the incidences of melanoma and lung are increasing over time while oral/pharynx remains steady. This could be part of why the previous multivariate model did not perform well in the later study years. For the 7718 diagnosed cases of oral/pharynx in South Carolina for these study years, there are approximately 168 (range: 19–651) cases per county per year leading to a rate of disease equal to 0.0001. Similarly, there were 20,156 and 44,595 diagnosed cases of melanoma and lung with an average of 438 (range: 25–2726) and 969 (range: 115–3885) cases per county per year leading to rates of disease equal to 0.0003 and 0.0008, respectively. These rates of disease are calculated as the total incidence over the entire study time divided by the total population at risk over the entire study time.

**Figure 2:**
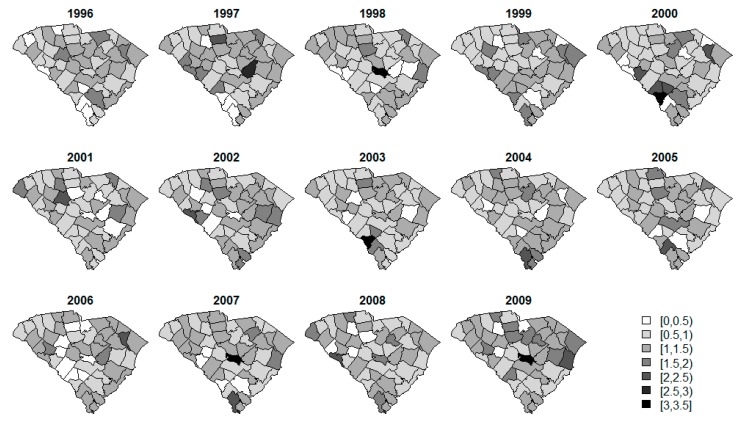
SIR of oral cavity and pharynx cancer.

**Figure 3:**
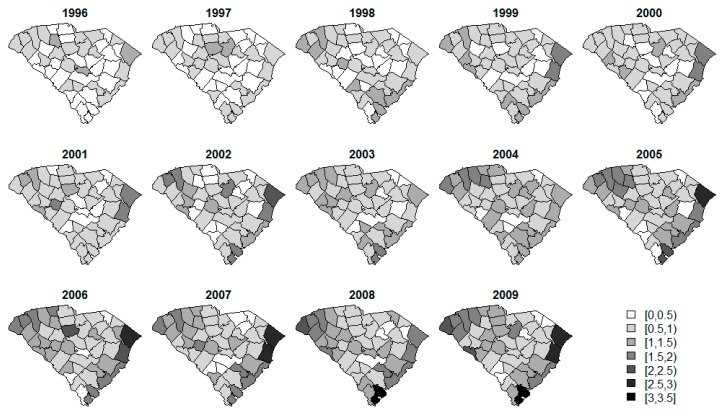
SIR of melanoma cancer of the skin.

**Figure 4:**
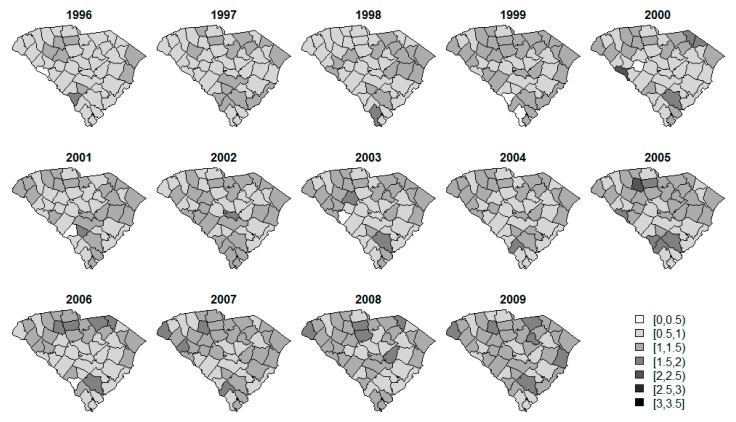
SIR of lung and bronchus cancer.

Standardized incidence rate (SIR) calculations are often a first step in data analysis. The SIRs per county over time for each of the diseases are displayed in Figure 2, Figure 3 and Figure 4. The SIR was calculated as a ratio of the observed cancer incidences to the expected rates of disease per county [20]. For interpretation purposes, a SIR of one indicates that the observed incidence is equal to that of the expected count. The SIRs for oral/pharynx do present some spatial structure, but are somewhat scattered as well. melanoma has a clear presence in the eastern coastal counties and the rates appear to increase over time within the same spatial pattern. Like oral/pharynx, lung appears to have a spatial structure in its distribution, albeit weak, and, like melanoma, the rates appear to increase over time.

The predictors for this analysis are demographic and environmental measures obtained from a variety of sources with known oral/pharynx, melanoma, and lung, as well as general oncological associations [4,5,6,21,22,23,24,25,26,27]. The demographic predictors come from the Area Health Resources Files dataset [28] while the environmental predictors come from the National Oceanic and Atmospheric Administration [29], South Carolina Department of Health and Environmental Control (SCDHEC) [30], and the North America Land Data Assimilation System [31]. 

For these methods, it is important to note which of these variables are spatial, temporal, or, S.T.; and predictors were designated as such based on either their availability or apparent amount of spatial and/or temporal variation. The proportion of persons with health insurance (pHI), median household radon level (radon), and proportion of African American population (pAA) are spatial where the two socioeconomic covariates are census data from the year 2000 and the measure of radon is a county-level average of in-home test kit results analyzed by the SCDHEC laboratory. The single temporal predictor is statewide average annual rainfall. Finally, the ST predictors are average daily sunlight (sun), unemployment rate of those 16 years or older (UER), and the proportion of persons in poverty (pppov). Note that the predictors are standardized for model-fitting purposes. Additionally, some of these variables are correlated, but each do still offer their own attributes for our risk model. For example, rainfall and statewide average sunlight are correlated, but rainfall could also offer information about humidity.

### 2.2. Statistical Methods

Here, we describe the methodology associated with the bivariate and multivariate mixture models utilized in this exploration. These methods are implemented via the R package R2WinBUGS which calls WinBUGS from R [32,33,34,35]. All models reached convergence with two chains for the Markov chain Monte Carlo sampler running for a total burn-in of 45,000 iterations and a sample of 5000. Trace plots of the deviance and some random effect standard deviations, as well as model BUGS code, are included in the supplemental materials.

We are interested in exploring the relationships between the two or three cancers in this bivariate or multivariate setting via a mixture model that was first introduced in Carroll et al. [36] as a form of model selection. These methods were then further improved and tested via a simulation study in Lawson et al. [2]. The general structure of this model for disease k in county i at time j is as follows:

$$\log\left(\theta_{ijk}\right)=\alpha_{0k}+\underset{h}{\sum }p_{ik}^{h}M_{ijk}^{h}$$

$$\underset{h}{\sum }p_{ik}^{h}=1;p_{ik}^{h}\in \left[0,1\right]$$

where $\alpha_{0k}\sim Norm\left(0,\tau_{a}^{-1}\right)$ is an intercept, $p_{ik}^{h}$ is a mixture parameter, and $M_{ijk}^{h}$ is a mixture component. In the previous study, the set of mixture parameters and components was given by h={S,ST}, thus containing a spatial and spatio-temporal parts. However, the temporal effect appeared to be causing model fit issues with oral/pharynx in the multivariate setting as this parameter was shared between diseases. Thus, in this paper we wish to explore the benefit of either keeping the same mixture component structure and allowing the temporal effect to vary between diseases or introducing a third mixture component for temporal effects. Hence, the three alternative mixture definitions for both the univariate and bivariate/multivariate cases are displayed in Table 2. The parameters displayed are such that $X_{i}$, $X_{j}$, and $X_{ij}$ are the $i^{th}$, $j^{th}$, and $ij^{th}$ values of the spatial, temporal, and ST covariates, respectively, $\beta \sim N\left(0,\tau_{\beta }^{-1}\right)$ for the general case are the fixed effect parameter estimates, $u_{ik}\sim N\left(0,\tau_{uk}^{-1}\right)$ is the uncorrelated spatial random effect. $v_{i}\sim N\left(\frac{1}{n_{i}}\underset{i\sim l}{\sum }v_{l},\frac{1}{n_{i}\tau_{v}}\right)$ is a correlated spatial random effect following an intrinsic conditional autoregressive (CAR) model, where $n_{i}$ is the number of neighbors for county i, and i~l indicates that the two counties i and l are neighbors (i≠l) [37,38]. $\gamma_{j}\sim N\left(\gamma_{j-1},\tau_{\gamma }^{-1}\right)$ is the temporal random effect modeled by a temporal random walk prior, $\phi_{ijk}\sim N\left(0,\tau_{\phi_{k}}^{-1}\right)$ is the uncorrelated ST interaction term, and all precisions are defined such that $\tau^{-1/2}\sim Unif\left(0,C\right)$. The random effects included here are typical of ST models such as the commonly applied Knorr-Held model [13,14]. Finally, the mixture parameter is made up of two parameters, $z_{i}$ and $a_{i}$ (univariate) or $z_{ik}$ and $a_{ik}$ (multivariate), that are correlated and uncorrelated in space, respectively. However, when three mixture components and parameters are needed, as in Alternative 2 (Alt2), constraints must be imposed such that the sum of the three mixture parameters is equaled to one. This is accomplished with the following structure: $\mathrm{logit}\left(q_{i}^{h}\right)=z_{i}^{h}+a_{i}^{h}$ and $p_{i}^{h}=q_{i}^{h}/\underset{h}{\sum }q_{i}^{h}$ or $\mathrm{logit}\left(q_{ik}^{h}\right)=z_{ik}^{h}+a_{ik}^{h}$ and $p_{ik}^{h}=q_{ik}^{h}/\underset{h}{\sum }q_{ik}^{h}$, where h={S,T,ST}.

The two correlated random effects, $v_{i}$ and $\gamma_{j}$, are defined such that they are common between diseases when there is no k subscript written. This is how information is shared between diseases of interest in these mixture models. Thus, Alternative 1 (Alt1) allows the temporally-correlated random effect to vary across diseases while the spatially-correlated random effect is shared amongst diseases. In the univariate setting, Alt1 is the same as F2PRED from the previous study [2], and the multivariate version of F2PRED offered the best fit for oral/pharynx. On the other hand, Alt2 has the interpretation that the temporally correlated effect is common between diseases, but the relationship between disease and temporal effect is different depending on the cancer of interest through the addition of $p_{ik}^{T}$. Finally, Alternative 3 (Alt3) is like Alt1, but it allows for a shared temporal random effect that is scaled by a function which is related to the disease of interest. Alt3 was only applied in the bivariate and multivariate settings as an attempt to further improve model fits by offering even more temporal flexibility. The two functions, leading to Alt3a and Alt3b, are such that:

$$\mathrm{Alt}3a:f_{k}\left(\gamma_{j},\rho_{k}\right)=\rho_{k}\gamma_{k},\rho_{k}\sim Norm\left(0,\tau_{\rho k}\right)$$

$$\mathrm{Alt}3b:f_{k}\left(\gamma_{j},\rho_{k}\right)={\left(\gamma_{j}\right)}^{\rho_{k}},\rho_{k}\sim Gamma\left(2,1\right)$$

Thus, Alt3a offers a scaling coefficient that differs per disease to relate to the temporal random effect while Alt3b offers a power term that differs per disease for relating to the temporal random effect. These alternatives offer different ways of incorporating more model flexibility relating to the temporal random effect. For notation, a “U,” “B,” or “M” following Alt1, Alt2, Alt3a, or Alt3b indicates that these models are fit in the univariate (each disease fitted separately), bivariate (only considers oral/pharynx with lung), or multivariate setting. 

## 3. Results

Table 3 presents the goodness of fit results for oral/pharynx with the models described in Section 2. Supplemental Tables S1–S3 present results for melanoma and lung as well as the overall model fit. The measurements here include Watanabe-Akaike information criterion (WAIC) and mean squared predictive error (MSPE). Both of these estimates indicate a superior model with a lower value. WAIC was proposed by Watanabe et al. as an alternative to the deviance information criterion and proves to be a better option of model assessment for mixture models [39,40]. These results indicate that Alt2B offers the best fit for oral/pharynx in terms of WAIC for all year classifications as well as MSPE for most of the year classifications. When examining WAIC for the multivariate models, there is evidence of improvement over the univariate models when comparing the results associated with Alt2. Further, the MSPE values are very large for the multivariate setting while the WAIC estimates remain fairly stable. This is largely driven by oral/pharynx indicating that prediction is poor, particularly for the final four years of the study period under these modeling scenarios. The MSPE estimates that only consider the first 10 years show that the multivariate models do perform best up to that point. Finally, these estimates demonstrate that Alt1, Alt2, and Alt3 show improvements over F2PRED for oral/pharynx with respect to multivariate model fit and model prediction, respectively.

Supplemental Figure S1 displays the temporal random effect ($\gamma_{j}$ or $\gamma_{jk}$) estimates from univariate and multivariate fits of Alt1 and Alt2 and the $f\left(\gamma_{j},\rho_{k}\right)$ estimates for Alt3aM and Alt3bM. Note that this parameter is shared between all diseases in bivariate and multivariate versions of Alt2, Alt3a, and Alt3b and the figure only displays the estimate associated with oral/pharynx for Alt1B and Alt1M. The estimates from Alt2U, Alt1B, Alt3bB, and Alt3bM appear to be essentially zero for the entire study period. All of the multivariate models except Alt3bM show the estimates increasing over time with a dramatic increase at year 10 (2005) for Alt1M and Alt3aM. This is much alike the jumps that were present in the example from Lawson et al., thus allowing the temporal random effect to be disease specific does not appear to fix that issue. However, it is worth noting that the other diseases’ temporal random effect estimates are much larger in magnitude than that of oral/pharynx for Alt1M. Additionally, while this parameter is shared between all diseases in Alt2M and Alt3bM, as well as all of the bivariate fits, the large jump does not appear for those situations.

Supplemental Figures S2 and S3 display the uncorrelated and correlated heterogeneity terms that relate to oral cavity and pharynx cancer. Note here that the correlated random effect is shared between diseases for all bivariate and multivariate models. The pattern for the uncorrelated random effect appears to be similar across all model specifications, however, the variances associated with Alt2U and Alt3bM are much larger than for any of the other models. Alternatively, for Alt2U, the variance for the correlated random effect is smaller and appears to follow a different pattern compared to the other models while this is not the case for Alt3bM. In general, the variance of the spatially-correlated random effect is greater than that of the uncorrelated random effect; however, when only considering Alt2U, the ranges for $u_{i}$ and $v_{i}$ are nearly identical.

Supplemental Figures S4 and S5 display the oral/pharynx related mixture parameter estimates for Alt1, Alt2, and Alt3 in the univariate, bivariate, and multivariate settings as labeled. The results here vary widely for all models. The univariate setting of Alt1 strongly selects the spatial mixture component with mixture parameter estimate very close to 1. This selection indicates that a better fit may arise from only employing the spatial component of Alt1U, and this is the case as the WAIC estimate associated with this refit is 3222.13 with a pD of 28.25. On the other hand, the MSPE is not improved by only using the spatial component. The bivariate model results are all alike and around the 0.5 range indicating that an equal mixture between the spatial and ST components is best. The estimates associated with Alt1M, Alt3aM, and Alt3bM agree and show much more variation, indicating that the central part of the state is slightly better explained by the spatial mixture component while the counties along western and eastern borders are better explained by the ST mixture component. For the Alt2 parameterization, the univariate and bivariate setting appears to prefer an equal mixture of the different components as a large majority of the counties are in the range of 0.3 to 0.4. The multivariate setting of Alt2 appears to indicate some selection relating to each of the mixture components for different counties. Both multivariate settings appear to agree somewhat in that central counties indicate better explanation from the spatial component while the counties on the eastern and western borders indicate a better explanation from the ST component. It could be the case that this relationship is an influence from the more common melanoma and lung that is not necessarily true for oral/pharynx.

**Figure 5:**
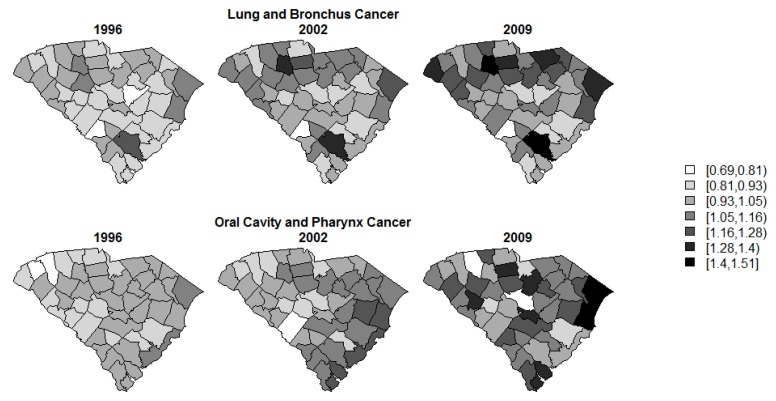
Overall posterior mean risk estimate ($\theta_{ij})$ for lung and bronchus cancers, as well as oral cavity and pharynx cancer from model Alt2B for a selection of years.

Since our goal is, ultimately, to improve the modeling of risk related to oral/pharynx cancer, Figure 5 displays the overall risk ($\theta_{ij}$) of lung and oral/pharynx associated with the best model, Alt2B. The fixed effect parameter estimates used for this calculation are included in Supplemental Table S4. Only 1996, 2002, and 2009 are included for brevity. From this figure, it can be deduced that lung has high risk in the northern portion of the state, as well as a lone county of elevated risk in the southern tip. For oral/pharynx cancer, the overall risk is higher in the eastern counties, particularly for some along the borders. This could be some edge effect which occurs in models of this nature. Temporally, the risk for both diseases appears to be increasing slightly over the years represented. In general, the trend in risk appears to be consistent across both space and time.

## 4. Discussion

The multivariate modeling of diseases offers many benefits; however, issues can arise when models fail to offer the appropriate amount of flexibility or inappropriate diseases are combined. The results above illustrate the importance of choosing the best model, as well as the best set of diseases, to incorporate to gain the desired improvement in fit for the less common disease. In this case, the bivariate modeling combining oral/pharynx with lung furnishes the best fit. 

The example from the paper in which these methods are introduced simply removes the data for the years where the multivariate model does not perform well with respect to oral/pharynx. In this exploration, we have successfully produced a slight variation on their methods that allow the entire data to be used. This is important because removing data results in a loss of information, and that is not an ideal solution. Ultimately, the results indicate that bivariate rather than multivariate methods are ideal for producing the best fit associated with oral/pharynx across the entire study time. 

As far as interpretation is concerned, these models do offer some difficulties. For the best model, Alt2B, the ideal interpretation of the random component comes from the information provided in the term: $p_{ik}^{S}\left(u_{ik}+v_{i}\right)+p_{ik}^{T}\gamma_{j}+p_{ik}^{ST}\phi_{ijk}$. This estimate represents the total of the confounding adjusted for in the fitted model and is displayed in Supplemental Figure 4. From this estimate, it is clear that the overall risk unexplained by the fixed effects becomes more variable over the study period. Increased risk appears in the northwestern and northeastern corners of the state; these areas are in the mountains and along the coast respectively. Interpretation of the fixed effects is similar in that the measure of interest would be $p_{ik}^{T}\beta_{k}^{T}$ for the particular county and disease of interest. Beyond that, an interpretation of the mixture parameter can be ascertained wherein, for example, if a particular county in Alt2B has $p_{ik}^{S}=0.1,\;p_{ik}^{T}=0.5,$ and $p_{ik}^{ST}=0.4$, then 10% of the overall variability in the risk is explained by spatial effects, 50% is explained by temporal effects, and 40% is explained by ST effects. If the entire state and all diseases have a fairly low estimate for one of the mixture parameters, as with Alt1U for oral/pharynx, it might be of interest to fit the model without that component to determine if it is necessary for explaining the overall variability in the risk of disease.

There are several explanations regarding the poor fit of the multivariate model. The first is that, while melanoma is also a more prevalent disease than oral/pharynx with common risk factors, they may be too different as far as spatial and temporal patterns are concerned to aid in the modeling of oral/pharynx in these data. Additionally, the multivariate models add even more random effects of the same type, and this could be leading to identification issues and, thus, poor fitting models. This is an issue common to all mixture model structures; however, the hierarchical structure and prior dependence, even when uninformative choices are made, that is characteristic of these Bayesian methods minimizes this issue as much as possible. We have assumed non-informative priors in this example, but even stronger prior assumptions could continue to minimize the issue of identifiability. Finally, there could be collinearity between the fixed and random effects. This issue could also lead to poorly-fitting models, as well as inaccurate estimates of the effects. Some, or all, of these explanations could impact the individual estimates presented in this paper, but when considering the overall risk of disease, which is our main goal, the random effects take on the role of representing the confounding variables to potentially produce the optimal fit.

## 5. Conclusions

The best model for explaining the overall risk of oral/pharynx is Alt2B. This model includes separate spatial, temporal, and ST mixture components, meaning that it is important to separate the temporal and ST fixed and random effects. Further, this model only combines oral/pharynx with lung, and that suggests that melanoma is not the best disease choice for modeling with oral/pharynx. Alternatively, multivariate models Alt3b and Alt3a offer improved fits for lung and melanoma, respectively.

## Figures and Tables

**Table 1:** Number and percent of zeros and imputed data per threshold.

*Disease*	*Number (%) of Data in Each Threshold*		
	0	1–4	5–10
*Oral/pharynx*	16 (2.5%)	200 (31.1%)	190 (29.5%)
*Melanoma*	13 (2.0%)	128 (19.9%)	151 (23.4%)
*Lung*	0 (0.0%)	4 (0.6%)	15 (2.3%)

**Table 2:** Alternative mixture term definitions.

Model	Spatial	Temporal	Spatio-Temporal	Mixture Parameter
Univariate				
F2PRED	$p_{i}M_{i}^{S}$ $M_{i}^{S}=X_{i}^{\prime }\beta^{S}+u_{i}+v_{i}$	-	$\left(1-p_{i}\right)M_{ij}^{ST}$ $M_{ij}^{ST}=X_{ij}^{\prime }\beta_{j}^{ST}+X_{j}^{\prime }\beta^{T}+$ $\gamma_{j}+\phi_{ij}$	$\mathrm{logit}\left(p_{i}\right)=z_{i}+\alpha_{i}$ $z_{i}\sim CAR\left(\tau_{z}^{-1}\right)$ $\alpha_{i}\sim Norm\left(0,\tau_{\alpha }^{-1}\right)$
Alt1	$p_{i}M_{i}^{S}$ $M_{i}^{S}=X_{i}^{\prime }\beta^{S}+u_{i}+v_{i}$	-	$\left(1-p_{i}\right)M_{ij}^{ST}$ $M_{ij}^{ST}=X_{ij}^{\prime }\beta_{j}^{ST}+X_{j}^{\prime }\beta^{T}+$ $\gamma_{j}+\phi_{ij}$	$\mathrm{logit}\left(p_{i}\right)=z_{i}+\alpha_{i}$ $z_{i}\sim CAR\left(\tau_{z}^{-1}\right)$ $\alpha_{i}\sim Norm\left(0,\tau_{\alpha }^{-1}\right)$
Alt2	$p_{i}^{S}M_{i}^{S}$ $M_{i}^{S}=X_{i}^{\prime }\beta^{S}+u_{i}+v_{i}$	$p_{i}^{T}M_{j}^{T}$ $M_{j}^{T}=X_{j}^{\prime }\beta^{T}+\gamma_{j}$	$p_{i}^{ST}M_{ij}^{ST}$ $M_{ij}^{ST}=X_{ij}^{\prime }\beta_{j}^{ST}+\phi_{ij}$	$\mathrm{logit}\left(q_{i}^{h}\right)=z_{i}^{h}+a_{i}^{h}$ $z_{i}^{h}\sim CAR\left(\tau_{p}^{-1}\right)$ $a_{i}^{h}\sim N\left(0,\tau_{a^{h}}^{-1}\right)$ $p_{i}^{h}=q_{i}^{h}/\underset{h}{\sum }q_{i}^{h}$ h={S,T,ST}
Bivariate/Multivariate ^1^				
F2PRED	$p_{ik}M_{ik}^{S}$ $M_{ik}^{S}=X_{i}^{\prime }\beta_{k}^{S}+u_{ik}+v_{i}$	-	$\left(1-p_{ik}\right)M_{ijk}^{ST}$ $M_{ijk}^{ST}=X_{ij}^{\prime }\beta_{jk}^{ST}+X_{j}^{\prime }\beta_{k}^{T}+$ $\gamma_{j}+\phi_{ijk}$	$\mathrm{logit}\left(p_{ik}\right)=z_{ik}+\alpha_{ik}$ $z_{ik}\sim CAR\left(\tau_{zk}^{-1}\right)$ $\alpha_{ik}\sim Norm\left(0,\tau_{\alpha k}^{-1}\right)$
Alt1	$p_{ik}M_{ik}^{S}$ $M_{ik}^{S}=X_{i}^{\prime }\beta_{k}^{S}+u_{ik}+v_{i}$	-	$\left(1-p_{ik}\right)M_{ijk}^{ST}$ $M_{ijk}^{ST}=X_{ij}^{\prime }\beta_{jk}^{ST}+X_{j}^{\prime }\beta_{k}^{T}+$ $\gamma_{jk}+\phi_{ijk}$	$\mathrm{logit}\left(p_{ik}\right)=z_{ik}+\alpha_{ik}$ $z_{ik}\sim CAR\left(\tau_{zk}^{-1}\right)$ $\alpha_{ik}\sim Norm\left(0,\tau_{\alpha k}^{-1}\right)$
Alt2	$p_{ik}^{S}M_{ik}^{S}$ $M_{ik}^{S}=X_{i}^{\prime }\beta_{k}^{S}+u_{ik}+v_{i}$	$p_{ik}^{T}M_{jk}^{T}$ $M_{jk}^{T}=X_{j}^{\prime }\beta_{k}^{T}+\gamma_{j}$	$p_{ik}^{ST}M_{ijk}^{ST}$ $M_{ijk}^{ST}=X_{ij}^{\prime }\beta_{jk}^{ST}+\phi_{ijk}$	$\mathrm{logit}\left(q_{ik}^{h}\right)=z_{ik}^{h}+a_{ik}^{h}$ $z_{ik}^{h}\sim CAR\left(\tau_{pk}^{-1}\right)$ $a_{ik}^{h}\sim N\left(0,\tau_{a_{k}^{h}}^{-1}\right)$ $p_{ik}^{h}=q_{ik}^{h}/\underset{h}{\sum }q_{ik}^{h}$ h={S,T,ST}
Alt3a	$p_{ik}M_{ik}^{S}$ $M_{ik}^{S}=X_{i}^{\prime }\beta_{k}^{S}+u_{ik}+v_{i}$	-	$\left(1-p_{ik}\right)M_{ijk}^{ST}$ $M_{ijk}^{ST}=X_{ij}^{\prime }\beta_{jk}^{ST}+X_{j}^{\prime }\beta_{k}^{T}+$ $\rho_{k}\gamma_{\rho k}+\phi_{ijk}$	$\mathrm{logit}\left(p_{ik}\right)=z_{ik}+\alpha_{ik}$ $z_{ik}\sim CAR\left(\tau_{zk}^{-1}\right)$ $\alpha_{ik}\sim Norm\left(0,\tau_{\alpha k}^{-1}\right)$
Alt3b	$p_{ik}M_{ik}^{S}$ $M_{ik}^{S}=X_{i}^{\prime }\beta_{k}^{S}+u_{ik}+v_{i}$	-	$\left(1-p_{ik}\right)M_{ijk}^{ST}$ $M_{ijk}^{ST}=X_{ij}^{\prime }\beta_{jk}^{ST}+X_{j}^{\prime }\beta_{k}^{T}+$ ${\left(\gamma_{j}\right)}^{\rho_{k}}+\phi_{ijk}$	$\mathrm{logit}\left(p_{ik}\right)=z_{ik}+\alpha_{ik}$ $z_{ik}\sim CAR\left(\tau_{zk}^{-1}\right)$ $\alpha_{ik}\sim Norm\left(0,\tau_{\alpha k}^{-1}\right)$

^1^ Note that bivariate and multivariate expressions can be grouped together because the only difference in their structure is that k=1,2 for the former and k=1,2,3 for the latter.

**Table 3:** Goodness of fit results for oral cavity and pharynx cancer.^1^

*Measure*	*Years*	*Univariate*			*Bivariate ^2^*				*Multivariate ^2^*				
		F2PRED	Alt1	Alt2	Alt1	Alt2	Alt3a	Alt3b	F2PRED	Alt1	Alt2	Alt3a	Alt3b
*WAIC*	’96–‘09	3241.74	3239.09	4448.71	3196.02	**3192.77**	3194.97	**3192.18**	3431.50	3426.94	3520.64	3470.28	4184.67
	’96–‘05	2298.34	2296.40	3130.11	**2265.29**	**2265.94**	**2264.57**	**2262.46**	2285.45	2479.40	2322.88	2473.00	2954.48
	’06–‘09	943.40	942.69	1318.60	930.73	**926.83**	930.40	**929.72**	1146.05	1000.37	1197.76	997.28	1230.20
*pD*	’96–‘09	105.89	105.29	157.31	94.31	96.66	93.44	93.76	131.08	131.07	141.69	149.43	501.02
	’96–‘05	75.39	74.35	96.14	65.53	68.85	66.34	65.84	74.29	103.97	73.92	106.24	344.19
	’06–‘09	30.50	30.94	61.17	28.79	27.80	27.09	27.92	56.78	42.40	67.78	43.18	156.83
*MSPE*	’96–‘09	153.44	154.03	117.00	117.93	**111.64**	116.81	115.23	14,050.59	14,037.08	13,995.61	13,953.98	13,678.78
	’96–‘05	103.93	103.75	84.43	84.53	80.58	82.13	82.00	73.51	**71.79**	79.94	74.44	73.05
	’06–‘09	49.51	50.28	32.57	33.40	**31.06**	34.68	33.22	13,977.08	13,965.29	13,915.67	13,879.53	13,605.73

^1^ Values in bold indicate the model with superior fit for that measure and time period. If multiple measures are indicated as superior for the same measure and time period combination, they are statistically equivalent. ^2^ The bivariate and multivariate results are the goodness of fit estimates related specifically to oral/pharynx. Even though multiple diseases were fit, these measures can be calculated in a way such that they only pertain to the fit of the individual diseases.

## Supplementary Materials

The following are available online at www.mdpi.com/1660-4601/14/5/503/s1, Figure S1: $\gamma_{jk}$, $\gamma_{j}$, and $f\left(\gamma_{j},\;\rho_{k}\right)$ posterior mean estimates for univariate and multivariate fits of Alt1, Alt2, and Alt3 for oral cavity and pharynx cancer, Figure S2: Posterior mean $u_{i}$ estimates for Alt1 and Alt2 in the univariate and multivariate framework, Figure S3: Posterior mean $v_{i}$ estimates for Alt1 and Alt2 as well as univariate and multivariate, Figure S4: Alt1 oral cavity and pharynx cancer posterior mean mixture parameter estimates in the univariate, bivariate, and multivariate settings, Figure S5: Alt2 oral/pharynx posterior mean mixture parameter estimates ($p_{i}^{S}$, $p_{i}^{T}$, $p_{i}^{ST}$, $p_{ik}^{S}$, $p_{ik}^{T}$, and $p_{ik}^{ST}$) in the univariate, bivariate, and multivariate settings, Figure S6: Sum of the posterior mean random effects times posterior mean mixture parameters ($p_{ik}^{S}\left(u_{ik}+v_{i}\right)+p_{ik}^{T}\gamma_{j}+p_{ik}^{ST}\phi_{ijk}$) associated with oral cavity and pharynx cancer for Alt2B, Table S1: Overall goodness of fit results for all cancers, Table S2: Goodness of fit results for melanoma cancer of the skin, Table S3: Goodness of fit results for lung and bronchus cancer, Table S4: Posterior mean fixed effect coefficient estimates for the best fitting model, Alt2B.
