# Supplementary material for: Extensions to Multivariate Space Time Mixture Modeling of Small Area Cancer Data
Source: Int J Environ Res Public Health. 2017 May 9;14(5):503. doi: 10.3390/ijerph14050503 (PMC5451954; doi:10.3390/ijerph14050503)
Supplement: Supplementary file 1 [file ijerph-14-00503-s001.pdf]

Supplemental Convergence Information

Trace plots of the deviance for all models, as well as some of the random effects' standard deviations are below. The univariate results are for oral/pharynx cancer. The deviance is also broken down by disease for the bivariate and multivariate plots such that d1 is lung, d2 is oral/pharynx, and d3 is melanoma.

Univariate

Alt 1

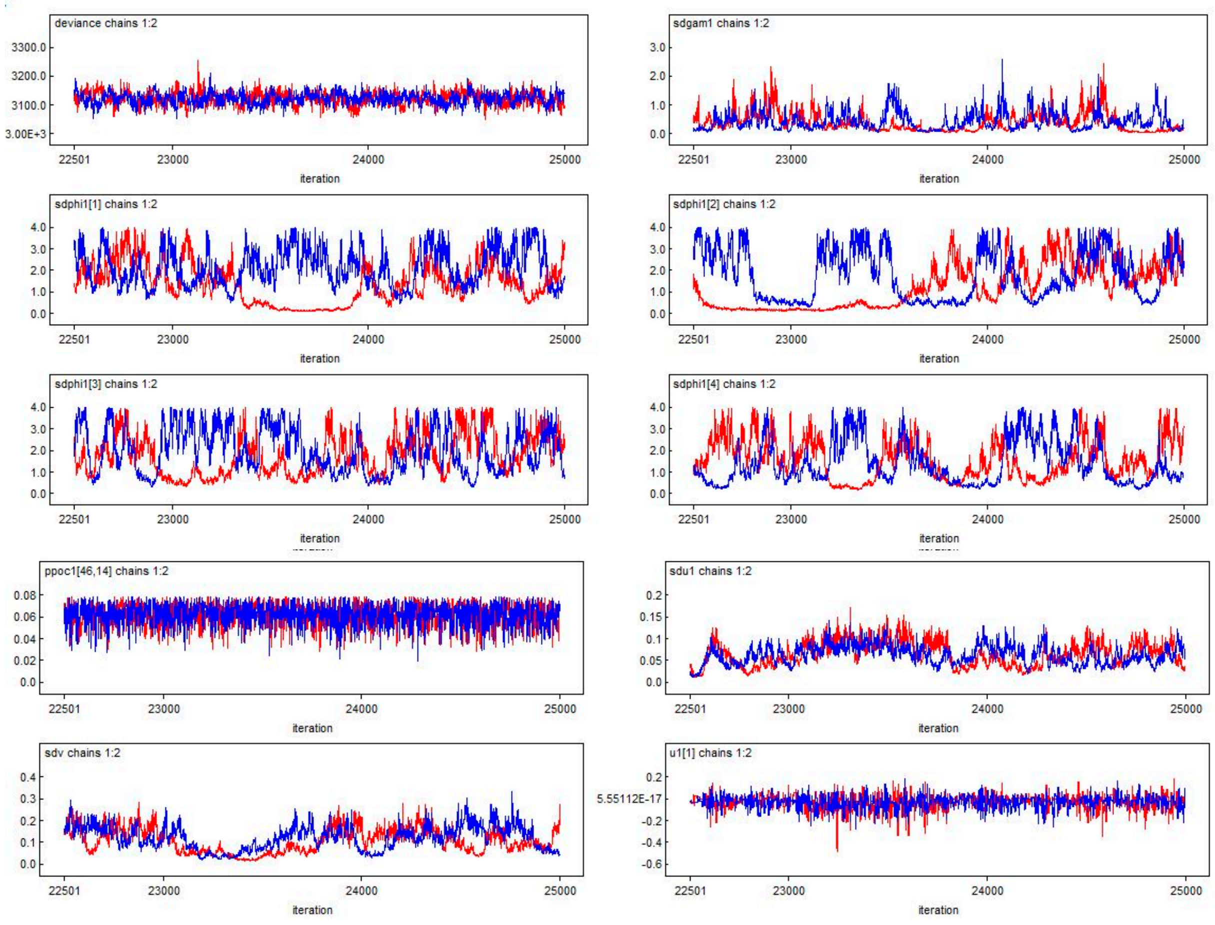

Alt 2

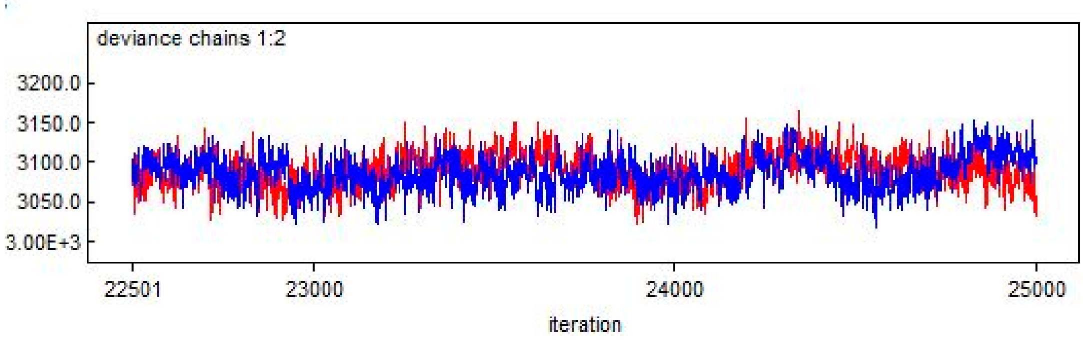

Bivariate

Alt 1

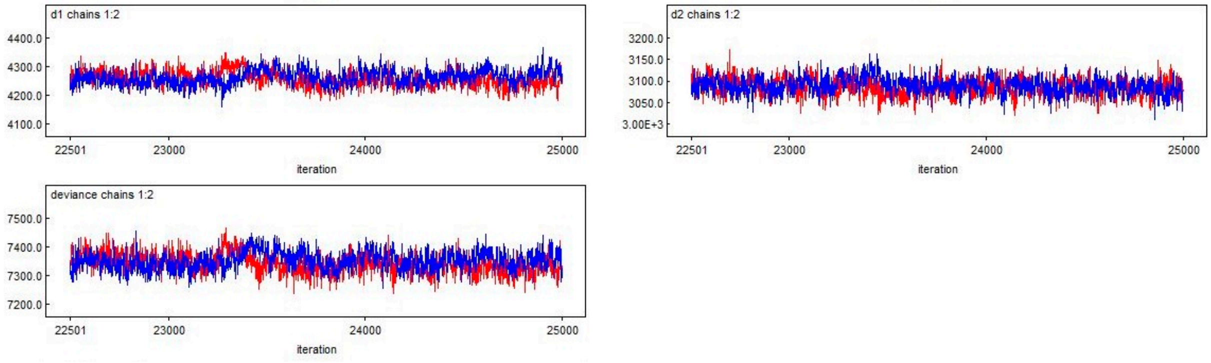

Alt2

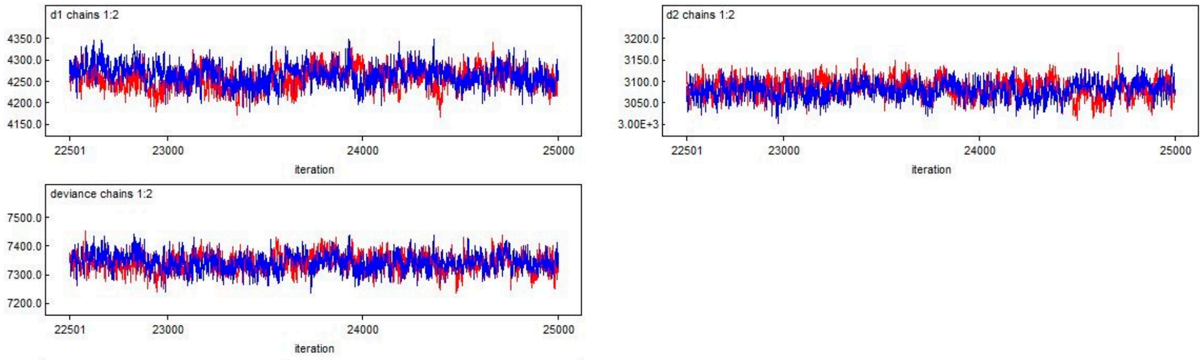

Alt 3a

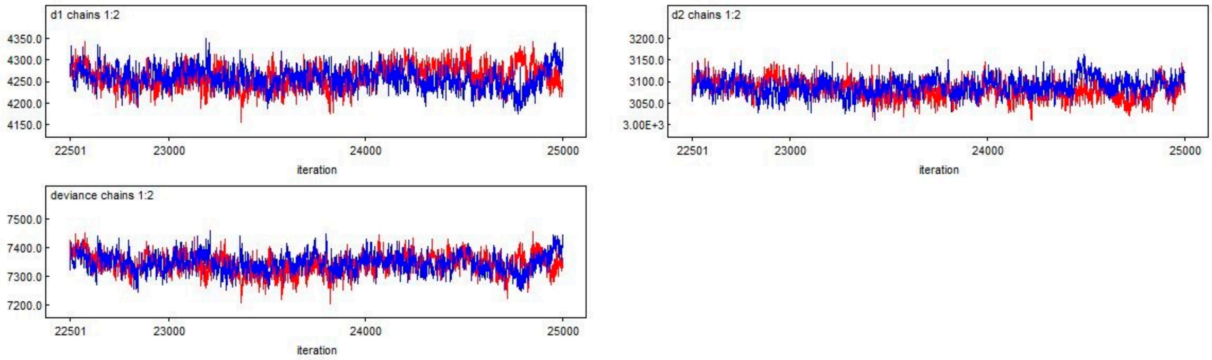

Alt 3b

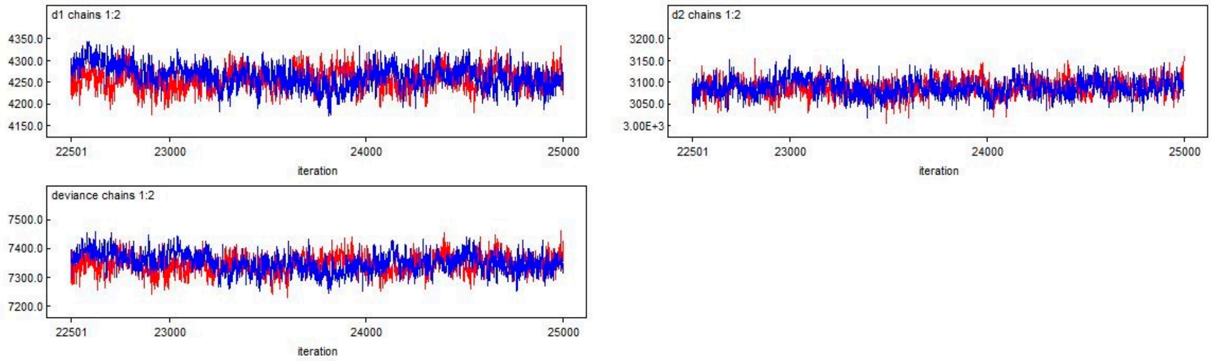

Multivariate

Alt 1

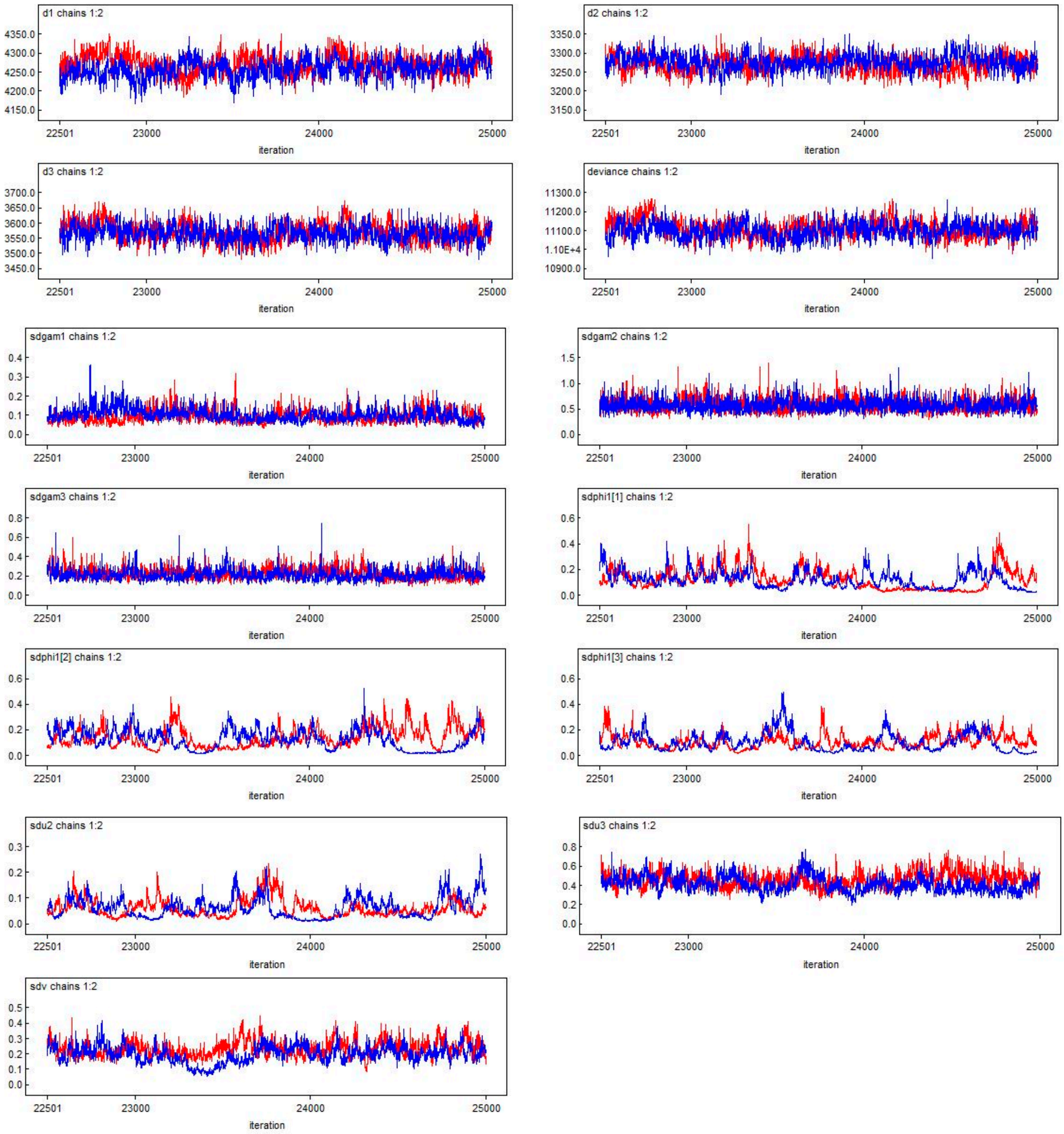

Alt 2

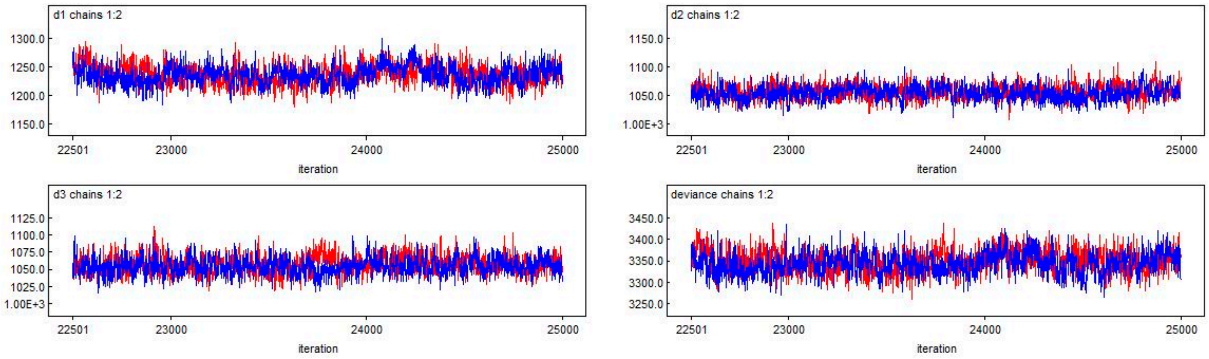

Alt 3a

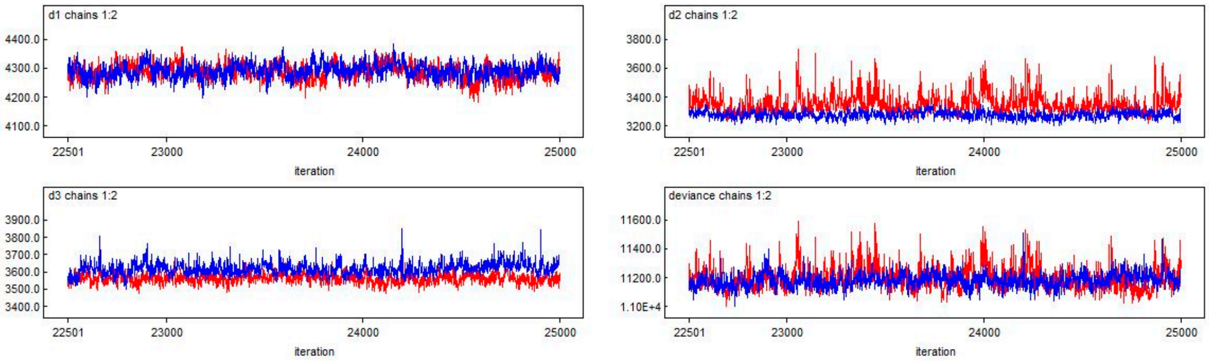
d1 chains 1:2
4400.0
4300.0
4200.0
4100.0
22501
23000
24000
25000
iteration
d2 chains 1:2
3800.0
3600.0
3400.0
3200.0
22501
23000
24000
25000
iteration
d3 chains 1:2
3900.0
3800.0
3700.0
3600.0
3500.0
3400.0
22501
23000
24000
25000
iteration
deviance chains 1:2
11600.0
11400.0
11200.0
1.10E+4
22501
23000
24000
25000
iteration

Alt 3b

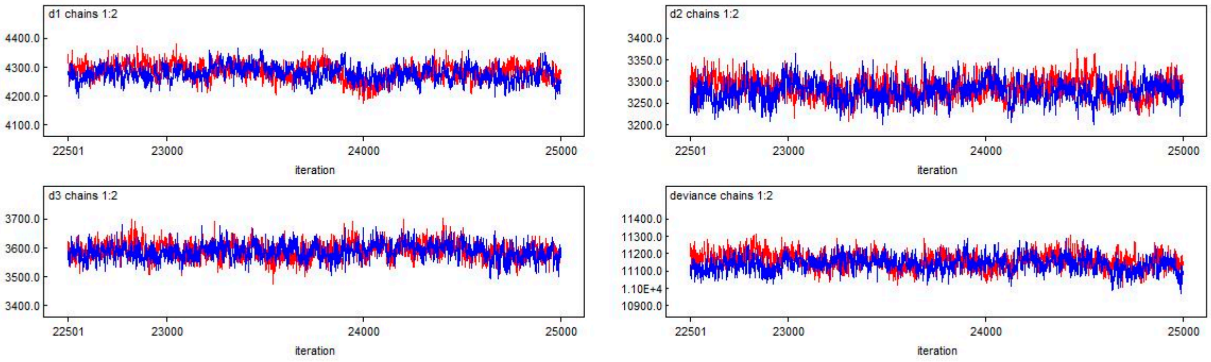
d1 chains 1:2
4400.0
4300.0
4200.0
4100.0
22501
23000
24000
25000
iteration
d2 chains 1:2
3400.0
3350.0
3300.0
3250.0
3200.0
22501
23000
24000
25000
iteration
d3 chains 1:2
3700.0
3600.0
3500.0
3400.0
22501
23000
24000
25000
iteration
deviance chains 1:2
11400.0
11300.0
11200.0
11100.0
1.10E+4
10900.0
22501
23000
24000
25000
iteration

Supplemental Tables and Figures

The following Tables S1–S3 display the goodness of fit measures for overall, lung, and melanoma over all models respectively.

Table S1. Overall goodness of fit results for all cancers.

		Univariate, Sum of Oral/Pharynx and Lung		Bivariate				Univariate, Sum of All 3		Multivariate			
		Alt1	Alt2	Alt1	Alt2	Alt3a	Alt3b	Alt1	Alt2	Alt1	Alt2	Alt3a	Alt3b
WAIC	’96–‘09	7695.81	8869.27	7615.10	**7609.03**	7618.74	**7611.42**	**11,494.82**	12,676.63	11,772.66	11,796.13	11,588.21	12,328.05
pD	’96–‘09	245.14	291.71	228.80	229.98	231.15	228.89	433.04	490.93	490.42	505.69	467.66	835.92
MSPE	’96–‘09	915.95	**735.50**	770.49	746.56	771.59	784.32	**1081.03**	934.77	14,975.96	14,807.69	14,809.43	14,425.79

Table S2. Goodness of fit results for melanoma cancer of the skin.

		Univariate		Multivariate			
		Alt1	Alt2	Alt1	Alt2	Alt3a	Alt3b
WAIC							
	’96–‘09	3799.01	3807.36	3839.02	3826.78	**3737.80**	3781.34
	’96–‘05	**2659.56**	2675.25	2689.43	2683.93	2672.38	2698.71
	’06–‘09	1139.45	1132.12	1137.36	1155.09	**1065.42**	1082.62
pD							
	’96–‘09	187.90	199.22	197.10	206.69	176.61	194.37
	’96–‘05	135.59	145.21	151.16	138.74	125.79	137.55
	’06–‘09	52.30	54.01	55.53	58.36	50.82	56.82
MSPE							
	’96–‘09	**165.08**	199.27	172.32	195.25	**164.75**	187.11
	’96–‘05	**103.88**	140.41	112.26	135.74	104.99	122.26
	’06–‘09	61.20	**58.86**	60.06	59.51	59.76	64.85

Table S3. Goodness of fit results for lung and bronchus cancer.

		Univariate		Bivariate				Multivariate			
		Alt1	Alt2	Alt1	Alt2	Alt3a	Alt3b	Alt1	Alt2	Alt3a	Alt3b
WAIC	'96–'09	4456.72	4420.56	4429.08	4416.26	4423.77	4419.24	4506.70	4448.71	4380.12	**4362.04**
	'96–'05	3131.58	3115.17	3116.14	3109.59	3115.81	3114.61	3136.12	3102.10	3113.24	**3096.35**
	'06–'09	1325.13	1305.40	1302.94	1306.66	1307.97	1304.63	1316.34	1302.10	**1266.89**	**1265.69**
pD	'96–'09	139.85	134.40	134.49	133.32	137.71	135.13	162.25	157.31	141.62	140.54
	'96–'05	81.93	81.97	81.16	81.90	82.68	82.20	82.20	82.20	100.17	98.85
	'06–'09	57.92	52.43	53.33	51.42	55.03	52.92	52.92	52.92	41.45	41.68
MSPE	'96–'09	761.92	618.50	652.56	634.92	654.78	669.09	766.56	616.84	690.71	**559.89**
	'96–'05	503.08	429.78	460.65	431.01	455.44	458.66	**511.05**	442.33	484.87	**392.09**
	'06–'09	258.84	188.73	191.91	203.91	247.26	210.44	255.51	174.51	205.83	**167.80**

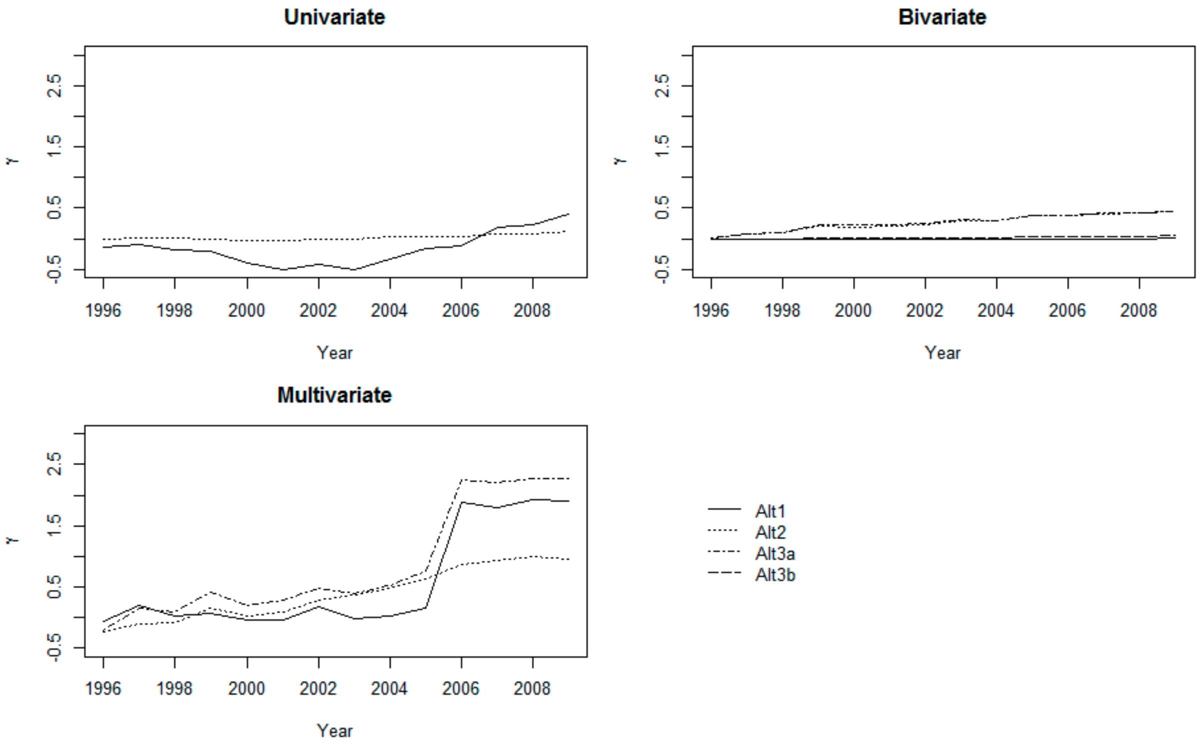

Figure S1. γ_{jk}, γ_j, and $f(\gamma_j, \rho_k)$ posterior mean estimates for univariate and multivariate fits of Alt1, Alt2, and Alt3 for oral cavity and pharynx cancer.

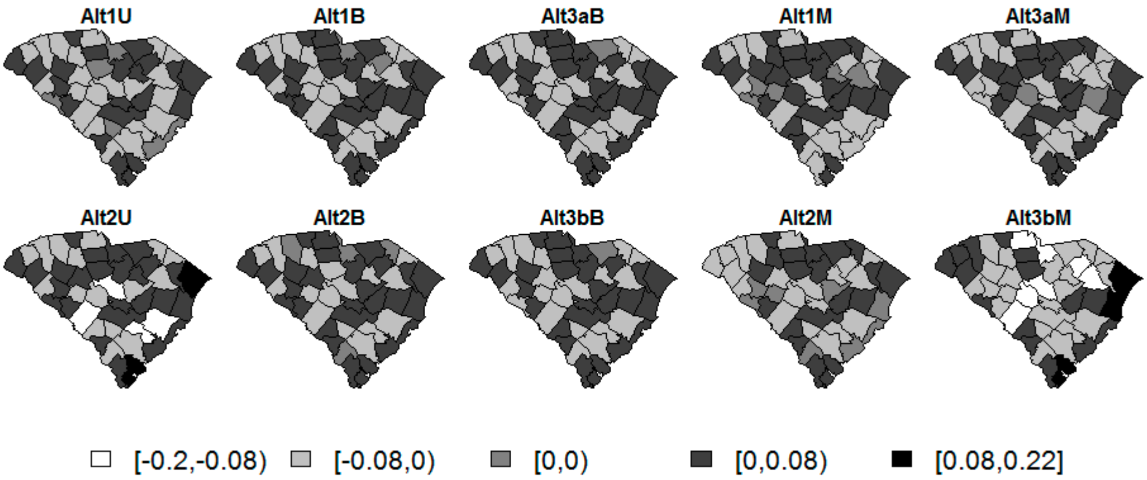

Figure S2. Posterior mean u_i estimates for Alt1 and Alt2 in the univariate and multivariate framework.

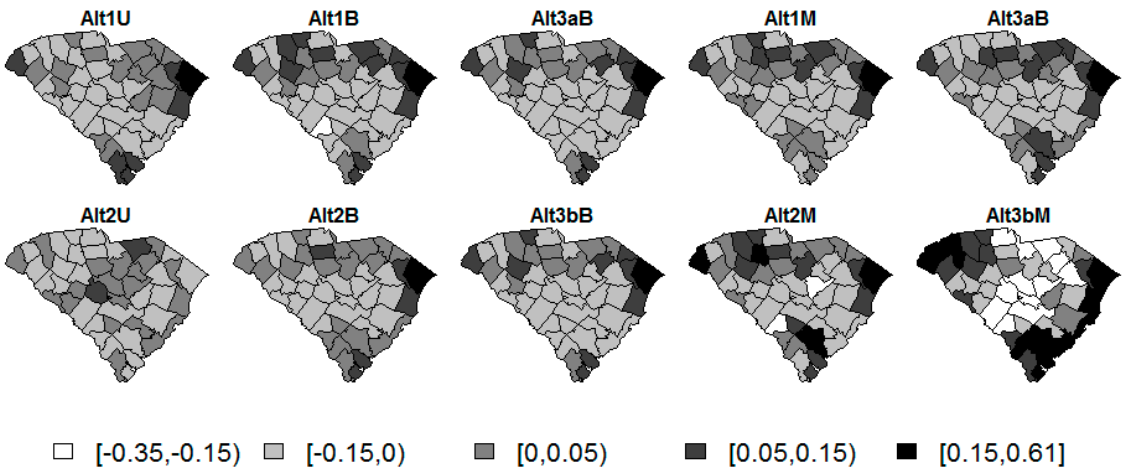

Figure S3. Posterior mean v_i estimates for Alt1 and Alt2 as well as univariate and multivariate.

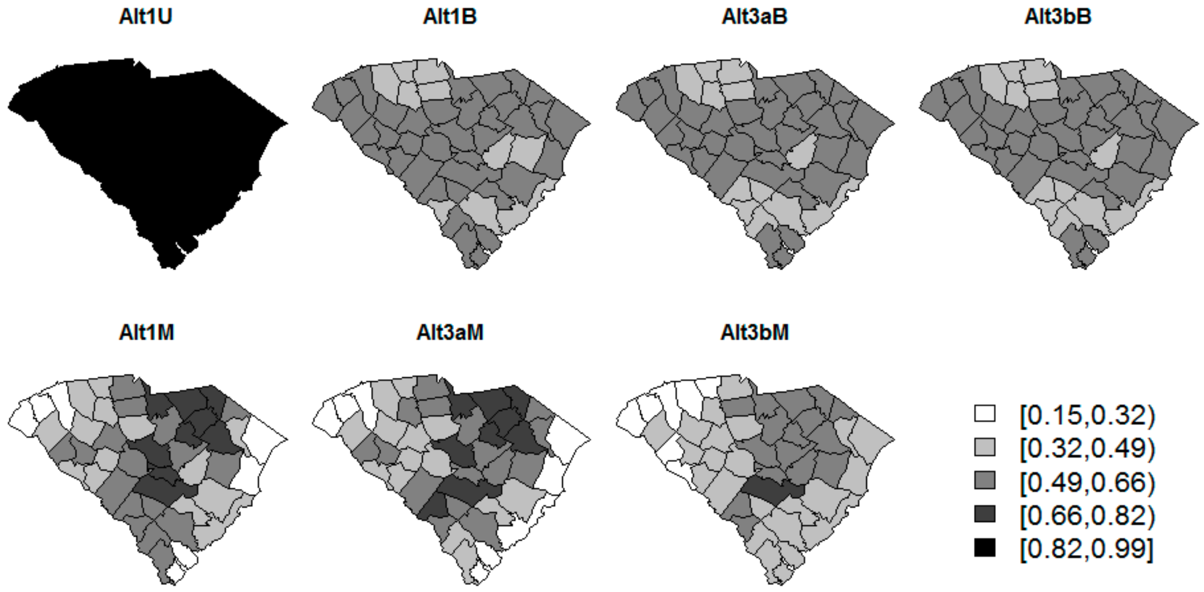

Figure S4. Alt1 oral cavity and pharynx cancer posterior mean mixture parameter estimates in the univariate, bivariate, and multivariate settings.

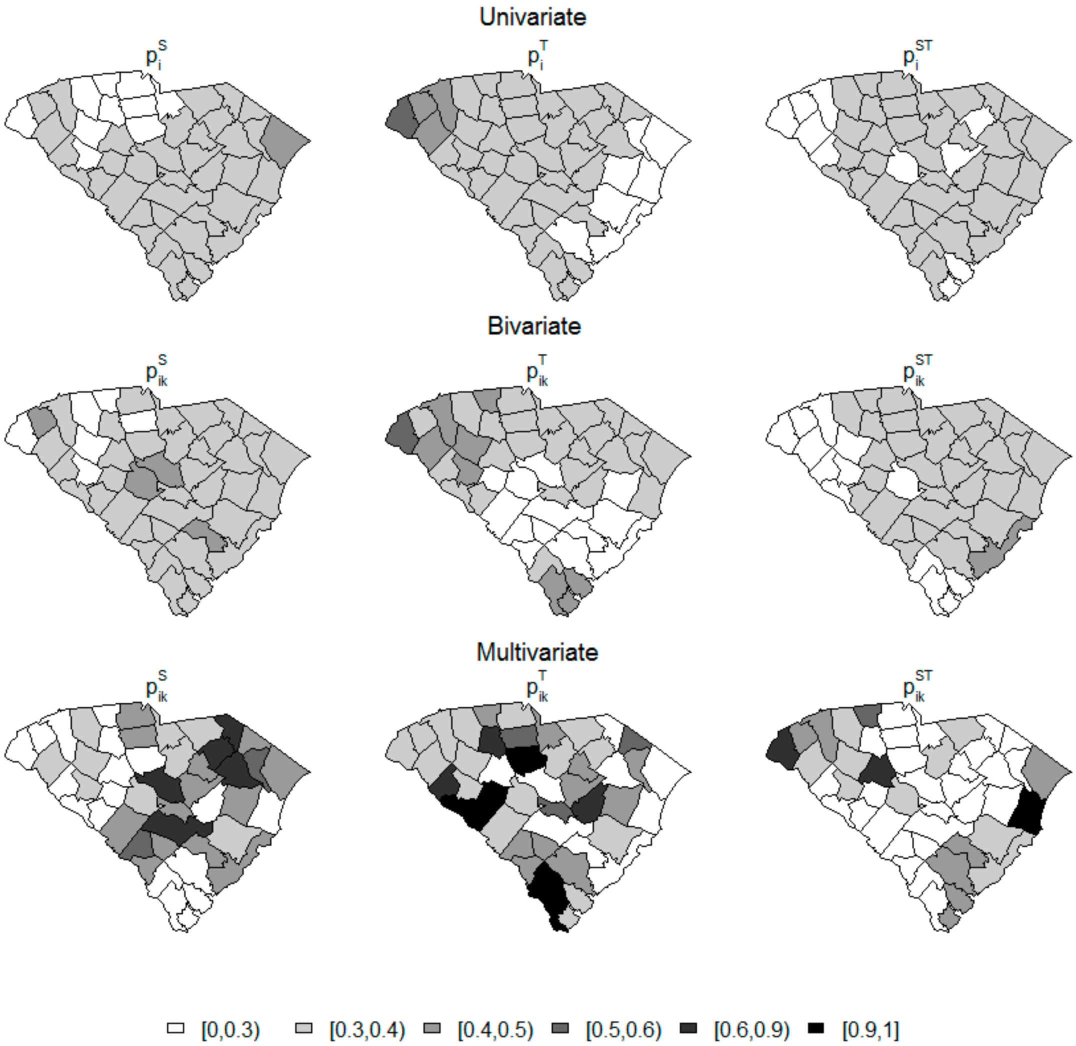

Figure S5. Alt2 oral/pharynx posterior mean mixture parameter estimates (p_i^S, p_i^T, p_i^{ST}, p_{ik}^S, p_{ik}^T, and p_{ik}^{ST}) in the univariate, bivariate, and multivariate settings.

Table S4. Posterior mean fixed effect coefficient estimates for the best fitting model, Alt2B.

Parameter	Mean	2.5%	97.5%
$\beta_{1,1}^S$	0.030819	−0.12271	0.203728
$\beta_{1,2}^S$	−0.02111	−0.1741	0.1677
$\beta_{1,3}^S$	−0.00107	−0.16281	0.1538
$\beta_{2,1}^S$	0.077557	−0.1123	0.2907
$\beta_{2,2}^S$	−0.16376	−0.43809	0.02683
$\beta_{2,3}^S$	0.080206	−0.11041	0.2881
β_1^T	−0.02091	−0.06491	0.018883
β_2^T	0.036927	−0.0375	0.1182
$\beta_{1,1,1}^{ST}$	−0.01605	−0.13121	0.061783
$\beta_{1,1,2}^{ST}$	0.013176	−0.05502	0.113015
$\beta_{1,1,3}^{ST}$	0.014457	−0.06366	0.117518
$\beta_{1,1,4}^{ST}$	−0.00554	−0.10001	0.077849
$\beta_{1,1,5}^{ST}$	0.01349	−0.06014	0.1165
$\beta_{1,1,6}^{ST}$	0.019758	−0.04797	0.126813
$\beta_{1,1,7}^{ST}$	0.00132	−0.07664	0.086639

$\beta^{ST}_{1,1,8}$	–0.01403	–0.10851	0.054963
$\beta^{ST}_{1,1,9}$	–0.00505	–0.09051	0.066932
$\beta^{ST}_{1,1,10}$	0.014129	–0.06109	0.115305
$\beta^{ST}_{1,1,11}$	–0.01349	–0.1151	0.057241
$\beta^{ST}_{1,1,12}$	–0.013	–0.10792	0.059841
$\beta^{ST}_{1,1,13}$	0.016244	–0.059	0.126113
$\beta^{ST}_{1,1,14}$	0.018128	–0.05227	0.1194
$\beta^{ST}_{1,2,1}$	–0.00153	–0.06074	0.054125
$\beta^{ST}_{1,2,2}$	0.008101	–0.03138	0.075319
$\beta^{ST}_{1,2,3}$	0.002473	–0.04271	0.063052
$\beta^{ST}_{1,2,4}$	0.003228	–0.04446	0.05606
$\beta^{ST}_{1,2,5}$	–0.00214	–0.05152	0.05026
$\beta^{ST}_{1,2,6}$	0.003596	–0.0421	0.064112
$\beta^{ST}_{1,2,7}$	0.005881	–0.03408	0.067362
$\beta^{ST}_{1,2,8}$	–0.00212	–0.06005	0.04479
$\beta^{ST}_{1,2,9}$	–0.0166	–0.09529	0.019074
$\beta^{ST}_{1,2,10}$	0.003702	–0.04558	0.06968
$\beta^{ST}_{1,2,11}$	–0.00713	–0.07584	0.036534
$\beta^{ST}_{1,2,12}$	–0.00947	–0.08793	0.0347
$\beta^{ST}_{1,2,13}$	–0.00921	–0.09105	0.036513
$\beta^{ST}_{1,2,14}$	0.003427	–0.04512	0.06183
$\beta^{ST}_{1,3,1}$	0.012069	–0.0942	0.146218
$\beta^{ST}_{1,3,2}$	–0.01506	–0.1226	0.079462
$\beta^{ST}_{1,3,3}$	0.002684	–0.1073	0.117008
$\beta^{ST}_{1,3,4}$	–0.0209	–0.1437	0.067091
$\beta^{ST}_{1,3,5}$	0.048153	–0.03964	0.222
$\beta^{ST}_{1,3,6}$	–0.01909	–0.14021	0.08059
$\beta^{ST}_{1,3,7}$	0.001756	–0.1052	0.108913
$\beta^{ST}_{1,3,8}$	–0.02004	–0.1352	0.06988
$\beta^{ST}_{1,3,9}$	0.042302	–0.04044	0.187503
$\beta^{ST}_{1,3,10}$	0.033306	–0.04981	0.1691
$\beta^{ST}_{1,3,11}$	–0.00688	–0.12351	0.1031
$\beta^{ST}_{1,3,12}$	0.012898	–0.0857	0.136613
$\beta^{ST}_{1,3,13}$	0.020966	–0.07667	0.169603
$\beta^{ST}_{1,3,14}$	–0.01256	–0.12871	0.087511
$\beta^{ST}_{2,1,1}$	0.010562	–0.1787	0.2209
$\beta^{ST}_{2,1,2}$	0.043124	–0.13	0.290205
$\beta^{ST}_{2,1,3}$	–0.05771	–0.3073	0.102505
$\beta^{ST}_{2,1,4}$	0.008362	–0.18223	0.201705
$\beta^{ST}_{2,1,5}$	0.07509	–0.0825	0.3578
$\beta^{ST}_{2,1,6}$	0.040369	–0.13681	0.262725
$\beta^{ST}_{2,1,7}$	0.039854	–0.13432	0.2703
$\beta^{ST}_{2,1,8}$	0.052673	–0.1228	0.318603
$\beta^{ST}_{2,1,9}$	–0.0162	–0.2189	0.156125
$\beta^{ST}_{2,1,10}$	–0.02632	–0.25393	0.147433
$\beta^{ST}_{2,1,11}$	0.051826	–0.09616	0.280005
$\beta^{ST}_{2,1,12}$	–0.05075	–0.26898	0.09944

$\beta^{ST}_{2,1,13}$	0.022996	−0.14102	0.229403
$\beta^{ST}_{2,1,14}$	0.02791	−0.15713	0.26839
$\beta^{ST}_{2,2,1}$	−0.00019	−0.12242	0.1247
$\beta^{ST}_{2,2,2}$	0.014576	−0.08649	0.16042
$\beta^{ST}_{2,2,3}$	0.018992	−0.08161	0.168015
$\beta^{ST}_{2,2,4}$	0.045032	−0.03691	0.2533
$\beta^{ST}_{2,2,5}$	0.024071	−0.06888	0.190618
$\beta^{ST}_{2,2,6}$	−0.00977	−0.14371	0.08895
$\beta^{ST}_{2,2,7}$	0.051478	−0.02805	0.2759
$\beta^{ST}_{2,2,8}$	−0.02054	−0.20052	0.083313
$\beta^{ST}_{2,2,9}$	0.014638	−0.07873	0.149015
$\beta^{ST}_{2,2,10}$	−0.02902	−0.2058	0.05692
$\beta^{ST}_{2,2,11}$	0.007344	−0.0882	0.130708
$\beta^{ST}_{2,2,12}$	−0.00346	−0.12452	0.1102
$\beta^{ST}_{2,2,13}$	−0.02393	−0.1796	0.065083
$\beta^{ST}_{2,2,14}$	0.008337	−0.1115	0.1573
$\beta^{ST}_{2,3,1}$	−0.05394	−0.30442	0.1208
$\beta^{ST}_{2,3,2}$	0.012937	−0.17571	0.233808
$\beta^{ST}_{2,3,3}$	−0.04308	−0.30924	0.1407
$\beta^{ST}_{2,3,4}$	−0.02199	−0.2552	0.1668
$\beta^{ST}_{2,3,5}$	0.03514	−0.14971	0.2894
$\beta^{ST}_{2,3,6}$	0.029794	−0.13982	0.26782
$\beta^{ST}_{2,3,7}$	0.019208	−0.1622	0.236208
$\beta^{ST}_{2,3,8}$	0.086876	−0.07877	0.412903
$\beta^{ST}_{2,3,9}$	−0.04227	−0.26351	0.1117
$\beta^{ST}_{2,3,10}$	−0.03697	−0.27001	0.1383
$\beta^{ST}_{2,3,11}$	0.01105	−0.16391	0.199333
$\beta^{ST}_{2,3,12}$	−0.0206	−0.2455	0.15421
$\beta^{ST}_{2,3,13}$	0.030659	−0.15302	0.268328
$\beta^{ST}_{2,3,14}$	0.013567	−0.1862	0.2403

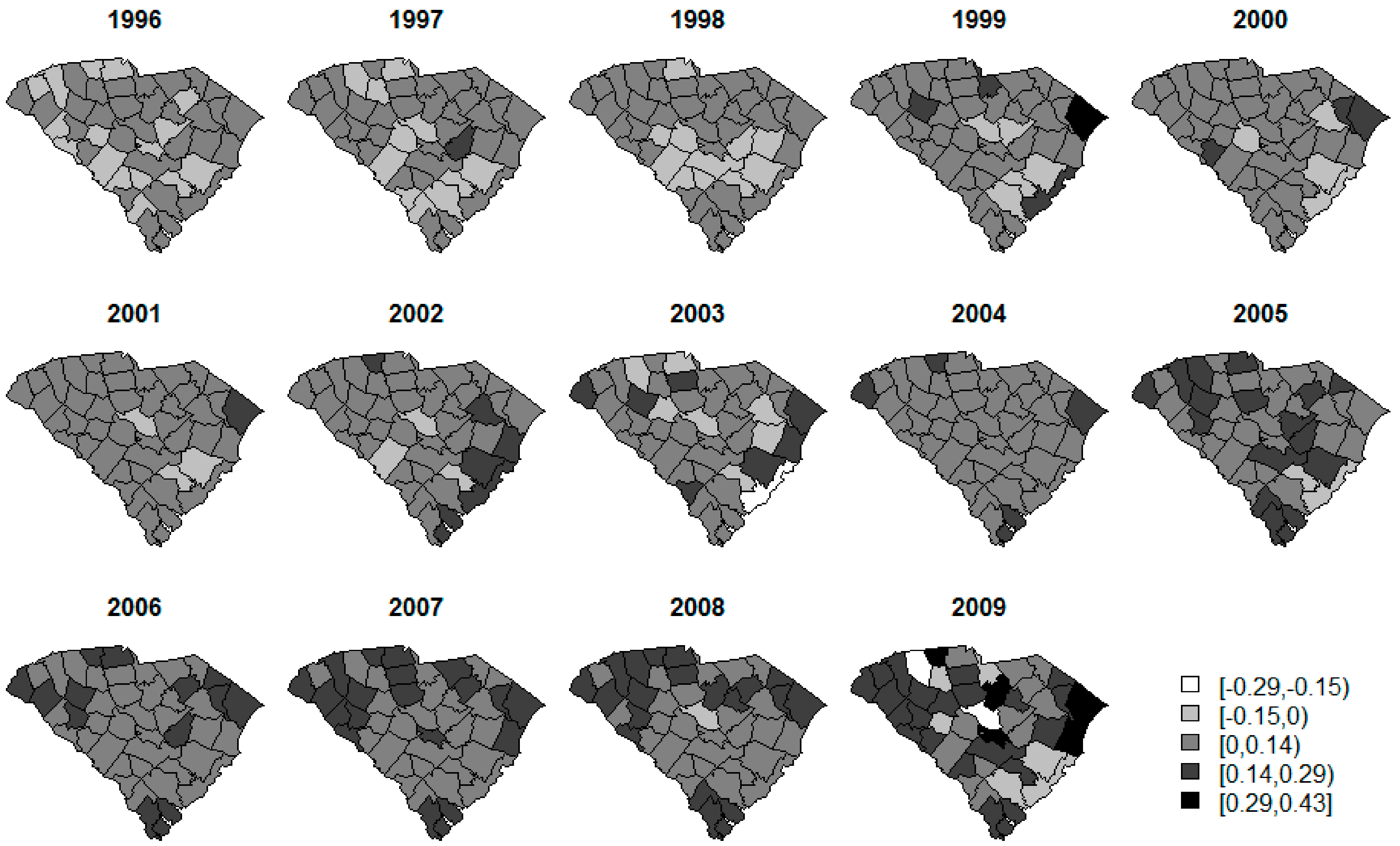

Figure S6. Sum of the posterior mean random effects times posterior mean mixture parameters ($p_{ik}^{S}(u_{ik}+v_i)+p_{ik}^{T}\gamma_j+p_{ik}^{ST}\phi_{ijk}$) associated with oral cavity and pharynx cancer for Alt2B.

Supplemental Code

Only multivariate code is included but the univariate equivalents can be deduced from these.

```
#Model Alt 1
model{
for (i in 1: 46){
        for (j in 1: 14){
#model set up
                exp1[i,j]<-POPL[i,j]*R1
                Y1[i,j]~dpois(mu1[i,j])
                log(mu1[i,j])<-log(exp1[i,j])+log(theta1[i,j])
                log(theta1[i,j])<-a01+p1[i]*modS1[i]+(1-p1[i])*modST1[i,j]
                ppoc1[i,j] <- exp(-mu1[i,j] + Y1[i,j]*log(mu1[i,j]) -
                        logfact(Y1[i,j]) )
                # inverse of the conditional predictive ordinate
                icpoc1[i,j] <- 1/ppoc1[i,j]
                logppoc1[i,j]<-log(ppoc1[i,j])
                ll1[i,j]<-Y1[i,j]*log(mu1[i,j])-logfact(Y1[i,j])-mu1[i,j]
                exp2[i,j]<-POPL[i,j]*R2
                Y2[i,j]~dpois(mu2[i,j])
                log(mu2[i,j])<-log(exp2[i,j])+log(theta2[i,j])
                log(theta2[i,j])<-a02+p2[i]*modS2[i]+(1-p2[i])*modST2[i,j]
                ppoc2[i,j] <- exp(-mu2[i,j] + Y2[i,j]*log(mu2[i,j]) -
                        logfact(Y2[i,j]) )
                # inverse of the conditional predictive ordinate
                icpoc2[i,j] <- 1/ppoc2[i,j]
                logppoc2[i,j]<-log(ppoc2[i,j])
                ll2[i,j]<-Y2[i,j]*log(mu2[i,j])-logfact(Y2[i,j])-mu2[i,j]
                exp3[i,j]<-POPL[i,j]*R3
                Y3[i,j]~dpois(mu3[i,j])
                log(mu3[i,j])<-log(exp3[i,j])+log(theta3[i,j])
                log(theta3[i,j])<-a03+p3[i]*modS3[i]+(1-p3[i])*modST3[i,j]
                ppoc3[i,j] <- exp(-mu3[i,j] + Y3[i,j]*log(mu3[i,j]) -
                        logfact(Y3[i,j]) )
                # inverse of the conditional predictive ordinate
                icpoc3[i,j] <- 1/ppoc3[i,j]
                logppoc3[i,j]<-log(ppoc3[i,j])
                ll3[i,j]<-Y3[i,j]*log(mu3[i,j])-logfact(Y3[i,j])-mu3[i,j]
#ST component
                modST1[i,j]<-gam1[j]+phi1[i,j]+aST1[1,j]*xST1[i,j]+aST1[2,j]*xST2[i,j]+
                        aST1[3,j]*xST3[i,j]+aT1*xT[j]
```

```
			modST2[i,j]<-gam2[j]+phi2[i,j]+aST2[1,j]*xST1[i,j]+aST2[2,j]*xST2[i,j]+
				aST2[3,j]*xST3[i,j]+aT2*xT[j]
			modST3[i,j]<-gam3[j]+phi3[i,j]+aST3[1,j]*xST1[i,j]+aST3[2,j]*xST2[i,j]+
				aST3[3,j]*xST3[i,j]+aT3*xT[j]
			phi1[i,j]~dnorm(0,tauphi1[j])
			phi2[i,j]~dnorm(0,tauphi2[j])
			phi3[i,j]~dnorm(0,tauphi3[j])
		}#close j loop
#spatial components
		modS1[i]<-v[i]+u1[i]+aS1[1]*xS1[i]+aS1[2]*xS2[i]+aS1[3]*xS3[i]
		modS2[i]<-v[i]+u2[i]+aS2[1]*xS1[i]+aS2[2]*xS2[i]+aS2[3]*xS3[i]
		modS3[i]<-v[i]+u3[i]+aS3[1]*xS1[i]+aS3[2]*xS2[i]+aS3[3]*xS3[i]
		u1[i]~dnorm(0,tauu1)
		u2[i]~dnorm(0,tauu2)
		u3[i]~dnorm(0,tauu3)
		logit(p1[i])<-z1[i]+alp1[i]
		alp1[i]~dnorm(0,taualp1)
		logit(p2[i])<-z2[i]+alp2[i]
		alp2[i]~dnorm(0,taualp2)
		logit(p3[i])<-z3[i]+alp3[i]
		alp3[i]~dnorm(0,taualp3)
}#close i loop
d1<--2*sum(ll1[,])
d2<--2*sum(ll2[,])
d3<--2*sum(ll3[,])
z1[1:46]~car.normal(adj[],weights[],num[],taup1)
z2[1:46]~car.normal(adj[],weights[],num[],taup2)
z3[1:46]~car.normal(adj[],weights[],num[],taup3)
v[1:46]~car.normal(adj[],weights[],num[],tauv)		#shared CH
for(k in 1:sumNumNeigh){weights[k]<-1}
for(j in 1:14){
		tauphi1[j]<-pow(sdphi1[j],-2)
		tauphi2[j]<-pow(sdphi2[j],-2)
		tauphi3[j]<-pow(sdphi3[j],-2)
		sdphi1[j]~dunif(0,4)
		sdphi2[j]~dunif(0,4)
		sdphi3[j]~dunif(0,4)
		MPLc1[j]<-sum(logppoc1[,j])
		MPLc2[j]<-sum(logppoc2[,j])
		MPLc3[j]<-sum(logppoc3[,j])
}#close j loop
gam1[1]~dnorm(0,taugam1)
```

```
gam2[1]~dnorm(0,taugam2)                                    #separate temporal
gam3[1]~dnorm(0,taugam3)
for (j in 2:14){
        gam1[j]~dnorm(gam1[j-1],taugam1)
        gam2[j]~dnorm(gam2[j-1],taugam2)
        gam3[j]~dnorm(gam3[j-1],taugam3)
        }#close j loop
taup1<-pow(sdp1,-2)
sdp1~dunif(0,4)
taup2<-pow(sdp2,-2)
sdp2~dunif(0,4)
taup3<-pow(sdp3,-2)
sdp3~dunif(0,4)
tauv<-pow(sdv,-2)
sdv~dunif(0,4)
taugam1<-pow(sdgam1,-2)
sdgam1~dunif(0,4)
taugam2<-pow(sdgam2,-2)
sdgam2~dunif(0,4)
taugam3<-pow(sdgam3,-2)
sdgam3~dunif(0,4)
a01~dnorm(0,tau01)
tau01<-pow(sd01,-2)
sd01~dunif(0,4)
a02~dnorm(0,tau02)
tau02<-pow(sd02,-2)
sd02~dunif(0,4)
a03~dnorm(0,tau03)
tau03<-pow(sd03,-2)
sd03~dunif(0,4)
tauu1<-pow(sdu1,-2)
sdu1~dunif(0,4)
tauu2<-pow(sdu2,-2)
sdu2~dunif(0,4)
tauu3<-pow(sdu3,-2)
sdu3~dunif(0,4)
taualp1<-pow(sdalp1,-2)
sdalp1~dunif(0,4)
taualp2<-pow(sdalp2,-2)
sdalp2~dunif(0,4)
taualp3<-pow(sdalp3,-2)
sdalp3~dunif(0,4)
```

```
aT1~dnorm(0,tauaT1)
tauaT1<-pow(sdaT1,-2)
sdaT1~dunif(0,4)
aT2~dnorm(0,tauaT2)
tauaT2<-pow(sdaT2,-2)
sdaT2~dunif(0,4)
aT3~dnorm(0,tauaT3)
tauaT3<-pow(sdaT3,-2)
sdaT3~dunif(0,4)
for (k in 1:3){
        aS1[k]~dnorm(0,tauaS1[k])
        tauaS1[k]<-pow(sdaS1[k],-2)
        sdaS1[k]~dunif(0,4)
        aS2[k]~dnorm(0,tauaS2[k])
        tauaS2[k]<-pow(sdaS2[k],-2)
        sdaS2[k]~dunif(0,4)
        aS3[k]~dnorm(0,tauaS3[k])
        tauaS3[k]<-pow(sdaS3[k],-2)
        sdaS3[k]~dunif(0,4)
        for (j in 1:14){
                aST1[k,j]~dnorm(0,tauaST1[k])
                aST2[k,j]~dnorm(0,tauaST2[k])
                aST3[k,j]~dnorm(0,tauaST3[k])
        }#close j loop
                tauaST1[k]<-pow(sdaST1[k],-2)
                sdaST1[k]~dunif(0,4)
                tauaST2[k]<-pow(sdaST2[k],-2)
                sdaST2[k]~dunif(0,4)
                tauaST3[k]<-pow(sdaST3[k],-2)
                sdaST3[k]~dunif(0,4)

}#close k loop
}#close model loop

#Model Alt 2
model{
        for (i in 1: 46){
        for (j in 1: 14){
#model set up
                exp1[i,j]<-POPL[i,j]*R1
                Y1[i,j]~dpois(mu1[i,j])
                log(mu1[i,j])<-log(exp1[i,j])+log(theta1[i,j])
```

```
            log(theta1[i,j])<-a01+p11[i]*modS1[i]+p12[i]*modT1[j]+p13[i]*modST1[i,j]
            ppoc1[i,j] <- exp(-mu1[i,j] + Y1[i,j]*log(mu1[i,j]) -
                  logfact(Y1[i,j]) )
            # inverse of the conditional predictive ordinate
            icpoc1[i,j] <- 1/ppoc1[i,j]
            logppoc1[i,j]<-log(ppoc1[i,j])
            ll1[i,j]<-Y1[i,j]*log(mu1[i,j])-logfact(Y1[i,j])-mu1[i,j]
            exp2[i,j]<-POPL[i,j]*R2
            Y2[i,j]~dpois(mu2[i,j])
            log(mu2[i,j])<-log(exp2[i,j])+log(theta2[i,j])
            log(theta2[i,j])<-a02+p21[i]*modS2[i]+p22[i]*modT2[j]+p23[i]*modST2[i,j]
            ppoc2[i,j] <- exp(-mu2[i,j] + Y2[i,j]*log(mu2[i,j]) -
                  logfact(Y2[i,j]) )
            # inverse of the conditional predictive ordinate
            icpoc2[i,j] <- 1/ppoc2[i,j]
            logppoc2[i,j]<-log(ppoc2[i,j])
            ll2[i,j]<-Y2[i,j]*log(mu2[i,j])-logfact(Y2[i,j])-mu2[i,j]
            exp3[i,j]<-POPL[i,j]*R3
            Y3[i,j]~dpois(mu3[i,j])
            log(mu3[i,j])<-log(exp3[i,j])+log(theta3[i,j])
            log(theta3[i,j])<-a03+p31[i]*modS3[i]+p32[i]*modT3[j]+p33[i]*modST3[i,j]
            ppoc3[i,j] <- exp(-mu3[i,j] + Y3[i,j]*log(mu3[i,j]) -
                  logfact(Y3[i,j]) )
            # inverse of the conditional predictive ordinate
            icpoc3[i,j] <- 1/ppoc3[i,j]
            logppoc3[i,j]<-log(ppoc3[i,j])
            ll3[i,j]<-Y3[i,j]*log(mu3[i,j])-logfact(Y3[i,j])-mu3[i,j]
            modST1[i,j]<-phi1[i,j]+aST1[1,j]*xST1[i,j]+aST1[2,j]*xST2[i,j]+
                  aST1[3,j]*xST3[i,j]
            modST2[i,j]<-phi2[i,j]+aST2[1,j]*xST1[i,j]+aST2[2,j]*xST2[i,j]+
                  aST2[3,j]*xST3[i,j]
            modST3[i,j]<-phi3[i,j]+aST3[1,j]*xST1[i,j]+aST3[2,j]*xST2[i,j]+
                  aST3[3,j]*xST3[i,j]
#ST RE
            phi1[i,j]~dnorm(0,tauphi1[j])
            phi2[i,j]~dnorm(0,tauphi2[j])
            phi3[i,j]~dnorm(0,tauphi3[j])
      }#close j loop
      modS1[i]<-v[i]+u1[i]+aS1[1]*xS1[i]+aS1[2]*xS2[i]+aS1[3]*xS3[i]
      modS2[i]<-v[i]+u2[i]+aS2[1]*xS1[i]+aS2[2]*xS2[i]+aS2[3]*xS3[i]
      modS3[i]<-v[i]+u3[i]+aS3[1]*xS1[i]+aS3[2]*xS2[i]+aS3[3]*xS3[i]
      u1[i]~dnorm(0,tauu1)
```

```
        u2[i]~dnorm(0,tauu2)                    #UH RE
        u3[i]~dnorm(0,tauu3)
        logit(q11[i])<-z11[i]+alp11[i]
        alp11[i]~dnorm(0,taualp1)
        logit(q12[i])<-z12[i]+alp12[i]
        alp12[i]~dnorm(0,taualp1)
        logit(q13[i])<-z13[i]+alp13[i]
        alp13[i]~dnorm(0,taualp1)
        qsum1[i]<-q11[i]+q12[i]+q13[i]
        p11[i]<-q11[i]/qsum1[i]
        p12[i]<-q12[i]/qsum1[i]
        p13[i]<-q13[i]/qsum1[i]
        logit(q21[i])<-z21[i]+alp21[i]
        alp21[i]~dnorm(0,taualp2)
        logit(q22[i])<-z22[i]+alp22[i]
        alp22[i]~dnorm(0,taualp2)
        logit(q23[i])<-z23[i]+alp23[i]
        alp23[i]~dnorm(0,taualp2)
        qsum2[i]<-q21[i]+q22[i]+q23[i]
        p21[i]<-q21[i]/qsum2[i]
        p22[i]<-q22[i]/qsum2[i]
        p23[i]<-q23[i]/qsum2[i]
        logit(q31[i])<-z31[i]+alp31[i]
        alp31[i]~dnorm(0,taualp3)
        logit(q32[i])<-z32[i]+alp32[i]
        alp32[i]~dnorm(0,taualp3)
        logit(q33[i])<-z33[i]+alp33[i]
        alp33[i]~dnorm(0,taualp3)
        qsum3[i]<-q31[i]+q32[i]+q33[i]
        p31[i]<-q31[i]/qsum3[i]
        p32[i]<-q32[i]/qsum3[i]
        p33[i]<-q33[i]/qsum3[i]
}#close i loop
d1<--2*sum(ll1[,])
d2<--2*sum(ll2[,])
d3<--2*sum(ll3[,])
z11[1:46]~car.normal(adj[],weights[],num[],taup1)
z12[1:46]~car.normal(adj[],weights[],num[],taup1)
z13[1:46]~car.normal(adj[],weights[],num[],taup1)
z21[1:46]~car.normal(adj[],weights[],num[],taup2)
z22[1:46]~car.normal(adj[],weights[],num[],taup2)
z23[1:46]~car.normal(adj[],weights[],num[],taup2)
```

```
z31[1:46]~car.normal(adj[],weights[],num[],taup3)
z32[1:46]~car.normal(adj[],weights[],num[],taup3)
z33[1:46]~car.normal(adj[],weights[],num[],taup3)
v[1:46]~car.normal(adj[],weights[],num[],tauv)                 #shared spatial
for(k in 1:sumNumNeigh){weights[k]<-1}
for(j in 1:14){
        modT1[j]<-gam[j]+aT1*xT[j]
        modT2[j]<-gam[j]+aT2*xT[j]                              #temporal component
        modT3[j]<-gam[j]+aT3*xT[j]
        tauphi1[j]<-pow(sdphi1[j],-2)
        tauphi2[j]<-pow(sdphi2[j],-2)
        tauphi3[j]<-pow(sdphi3[j],-2)
        sdphi1[j]~dunif(0,4)
        sdphi2[j]~dunif(0,4)
        sdphi3[j]~dunif(0,4)
        MPLc1[j]<-sum(logppoc1[,j])
        MPLc2[j]<-sum(logppoc2[,j])
        MPLc3[j]<-sum(logppoc3[,j])
}#close j loop
gam[1]~dnorm(0,taugam)
for (j in 2:14){
        gam[j]~dnorm(gam[j-1],taugam)                           #shared temporal RE
        }#close j loop
taup1<-pow(sdp1,-2)
sdp1~dunif(0,4)
taup2<-pow(sdp2,-2)
sdp2~dunif(0,4)
taup3<-pow(sdp3,-2)
sdp3~dunif(0,4)
tauv<-pow(sdv,-2)
sdv~dunif(0,4)
taugam<-pow(sdgam,-2)
sdgam~dunif(0,4)
a01~dnorm(0,tau01)
tau01<-pow(sd01,-2)
sd01~dunif(0,4)
a02~dnorm(0,tau02)
tau02<-pow(sd02,-2)
sd02~dunif(0,4)
a03~dnorm(0,tau03)
tau03<-pow(sd03,-2)
sd03~dunif(0,4)
```

```
tauu1<-pow(sdu1,-2)
sdu1~dunif(0,4)
tauu2<-pow(sdu2,-2)
sdu2~dunif(0,4)
tauu3<-pow(sdu3,-2)
sdu3~dunif(0,4)
taualp1<-pow(sdalp1,-2)
sdalp1~dunif(0,4)
taualp2<-pow(sdalp2,-2)
sdalp2~dunif(0,4)
taualp3<-pow(sdalp3,-2)
sdalp3~dunif(0,4)
aT1~dnorm(0,tauaT1)
tauaT1<-pow(sdaT1,-2)
sdaT1~dunif(0,4)
aT2~dnorm(0,tauaT2)
tauaT2<-pow(sdaT2,-2)
sdaT2~dunif(0,4)
aT3~dnorm(0,tauaT3)
tauaT3<-pow(sdaT3,-2)
sdaT3~dunif(0,4)
for (k in 1:3){
	aS1[k]~dnorm(0,tauaS1[k])
	tauaS1[k]<-pow(sdaS1[k],-2)
	sdaS1[k]~dunif(0,4)
	aS2[k]~dnorm(0,tauaS2[k])
	tauaS2[k]<-pow(sdaS2[k],-2)
	sdaS2[k]~dunif(0,4)
	aS3[k]~dnorm(0,tauaS3[k])
	tauaS3[k]<-pow(sdaS3[k],-2)
	sdaS3[k]~dunif(0,4)
	for (j in 1:14){
		aST1[k,j]~dnorm(0,tauaST1[k])
		aST2[k,j]~dnorm(0,tauaST2[k])
		aST3[k,j]~dnorm(0,tauaST3[k])
	}#close j loop
		tauaST1[k]<-pow(sdaST1[k],-2)
		sdaST1[k]~dunif(0,4)
		tauaST2[k]<-pow(sdaST2[k],-2)
		sdaST2[k]~dunif(0,4)
		tauaST3[k]<-pow(sdaST3[k],-2)
		sdaST3[k]~dunif(0,4)
```

```
}#close k loop
}#close model loop

#Model Alt 3a
model{
for (i in 1: 46){
        for (j in 1: 14){
                exp1[i,j]<-POPL[i,j]*R1
                Y1[i,j]~dpois(mu1[i,j])
                log(mu1[i,j])<-log(exp1[i,j])+log(theta1[i,j])
                log(theta1[i,j])<-a01+p1[i]*modS1[i]+(1-p1[i])*modST1[i,j]
                ppoc1[i,j] <- exp(-mu1[i,j] + Y1[i,j]*log(mu1[i,j]) -
                        logfact(Y1[i,j]) )
                # inverse of the conditional predictive ordinate
                icpoc1[i,j] <- 1/ppoc1[i,j]
                logppoc1[i,j]<-log(ppoc1[i,j])
                ll1[i,j]<-Y1[i,j]*log(mu1[i,j])-logfact(Y1[i,j])-mu1[i,j]
                exp2[i,j]<-POPL[i,j]*R2
                Y2[i,j]~dpois(mu2[i,j])
                log(mu2[i,j])<-log(exp2[i,j])+log(theta2[i,j])
                log(theta2[i,j])<-a02+p2[i]*modS2[i]+(1-p2[i])*modST2[i,j]
                ppoc2[i,j] <- exp(-mu2[i,j] + Y2[i,j]*log(mu2[i,j]) -
                        logfact(Y2[i,j]) )
                # inverse of the conditional predictive ordinate
                icpoc2[i,j] <- 1/ppoc2[i,j]
                logppoc2[i,j]<-log(ppoc2[i,j])
                ll2[i,j]<-Y2[i,j]*log(mu2[i,j])-logfact(Y2[i,j])-mu2[i,j]
                exp3[i,j]<-POPL[i,j]*R3
                Y3[i,j]~dpois(mu3[i,j])
                log(mu3[i,j])<-log(exp3[i,j])+log(theta3[i,j])
                log(theta3[i,j])<-a03+p3[i]*modS3[i]+(1-p3[i])*modST3[i,j]
                ppoc3[i,j] <- exp(-mu3[i,j] + Y3[i,j]*log(mu3[i,j]) -
                        logfact(Y3[i,j]) )
                # inverse of the conditional predictive ordinate
                icpoc3[i,j] <- 1/ppoc3[i,j]
                logppoc3[i,j]<-log(ppoc3[i,j])
                ll3[i,j]<-Y3[i,j]*log(mu3[i,j])-logfact(Y3[i,j])-mu3[i,j]
                modST1[i,j]<-rho[1]*gam[j]+phi1[i,j]+aST1[1,j]*xST1[i,j]+aST1[2,j]*xST2[i,j]+
                        aST1[3,j]*xST3[i,j]+aT1*xT[j]
                modST2[i,j]<-rho[2]*gam[j]+phi2[i,j]+aST2[1,j]*xST1[i,j]+aST2[2,j]*xST2[i,j]+
                        aST2[3,j]*xST3[i,j]+aT2*xT[j]
                modST3[i,j]<-rho[3]*gam[j]+phi3[i,j]+aST3[1,j]*xST1[i,j]+aST3[2,j]*xST2[i,j]+
```

```
                    aST3[3,j]*xST3[i,j]+aT3*xT[j]
                phi1[i,j]~dnorm(0,tauphi1[j])
                phi2[i,j]~dnorm(0,tauphi2[j])
                phi3[i,j]~dnorm(0,tauphi3[j])
        }#close j loop
        modS1[i]<-v[i]+u1[i]+aS1[1]*xS1[i]+aS1[2]*xS2[i]+aS1[3]*xS3[i]
        modS2[i]<-v[i]+u2[i]+aS2[1]*xS1[i]+aS2[2]*xS2[i]+aS2[3]*xS3[i]
        modS3[i]<-v[i]+u3[i]+aS3[1]*xS1[i]+aS3[2]*xS2[i]+aS3[3]*xS3[i]
        u1[i]~dnorm(0,tauu1)
        u2[i]~dnorm(0,tauu2)
        u3[i]~dnorm(0,tauu3)
        logit(p1[i])<-z1[i]+alp1[i]
        alp1[i]~dnorm(0,taualp1)
        logit(p2[i])<-z2[i]+alp2[i]
        alp2[i]~dnorm(0,taualp2)
        logit(p3[i])<-z3[i]+alp3[i]
        alp3[i]~dnorm(0,taualp3)
}#close i loop
d1<--2*sum(ll1[,])
d2<--2*sum(ll2[,])
d3<--2*sum(ll3[,])
z1[1:46]~car.normal(adj[],weights[],num[],taup1)
z2[1:46]~car.normal(adj[],weights[],num[],taup2)
z3[1:46]~car.normal(adj[],weights[],num[],taup3)
v[1:46]~car.normal(adj[],weights[],num[],tauv)
for(k in 1:sumNumNeigh){weights[k]<-1}
for(j in 1:14){
        tauphi1[j]<-pow(sdphi1[j],-2)
        tauphi2[j]<-pow(sdphi2[j],-2)
        tauphi3[j]<-pow(sdphi3[j],-2)
        sdphi1[j]~dunif(0,4)
        sdphi2[j]~dunif(0,4)
        sdphi3[j]~dunif(0,4)
        MPLc1[j]<-sum(logppoc1[,j])
        MPLc2[j]<-sum(logppoc2[,j])
        MPLc3[j]<-sum(logppoc3[,j])
}#close j loop
gam[1]~dnorm(0,taugam)
for (j in 2:14){
        gam[j]~dnorm(gam[j-1],taugam)
}#close j loop
taup1<-pow(sdp1,-2)
```

```
sdp1~dunif(0,4)
taup2<-pow(sdp2,-2)
sdp2~dunif(0,4)
taup3<-pow(sdp3,-2)
sdp3~dunif(0,4)
tauv<-pow(sdv,-2)
sdv~dunif(0,4)
taugam<-pow(sdgam,-2)
sdgam~dunif(0,4)
a01~dnorm(0,tau01)
tau01<-pow(sd01,-2)
sd01~dunif(0,4)
a02~dnorm(0,tau02)
tau02<-pow(sd02,-2)
sd02~dunif(0,4)
a03~dnorm(0,tau03)
tau03<-pow(sd03,-2)
sd03~dunif(0,4)
tauu1<-pow(sdu1,-2)
sdu1~dunif(0,4)
tauu2<-pow(sdu2,-2)
sdu2~dunif(0,4)
tauu3<-pow(sdu3,-2)
sdu3~dunif(0,4)
taualp1<-pow(sdalp1,-2)
sdalp1~dunif(0,4)
taualp2<-pow(sdalp2,-2)
sdalp2~dunif(0,4)
taualp3<-pow(sdalp3,-2)
sdalp3~dunif(0,4)
aT1~dnorm(0,tauaT1)
tauaT1<-pow(sdaT1,-2)
sdaT1~dunif(0,4)
aT2~dnorm(0,tauaT2)
tauaT2<-pow(sdaT2,-2)
sdaT2~dunif(0,4)
aT3~dnorm(0,tauaT3)
tauaT3<-pow(sdaT3,-2)
sdaT3~dunif(0,4)
for (k in 1:3){
	aS1[k]~dnorm(0,tauaS1[k])
	tauaS1[k]<-pow(sdaS1[k],-2)
```

```
		sdaS1[k]~dunif(0,4)
		aS2[k]~dnorm(0,tauaS2[k])
		tauaS2[k]<-pow(sdaS2[k],-2)
		sdaS2[k]~dunif(0,4)
		aS3[k]~dnorm(0,tauaS3[k])
		tauaS3[k]<-pow(sdaS3[k],-2)
		sdaS3[k]~dunif(0,4)
		rho[k]~dnorm(0,taurho[k])
		taurho[k]<-pow(sdrho[k],-2)
		sdrho[k]~dunif(0,10)
		for (j in 1:14){
			aST1[k,j]~dnorm(0,tauaST1[k])
			aST2[k,j]~dnorm(0,tauaST2[k])
			aST3[k,j]~dnorm(0,tauaST3[k])
		}#close j loop
			tauaST1[k]<-pow(sdaST1[k],-2)
			sdaST1[k]~dunif(0,4)
			tauaST2[k]<-pow(sdaST2[k],-2)
			sdaST2[k]~dunif(0,4)
			tauaST3[k]<-pow(sdaST3[k],-2)
			sdaST3[k]~dunif(0,4)

}#close k loop
}#close model loop

#Model Alt 3b
model{
for (i in 1: 46){
	for (j in 1: 14){
		exp1[i,j]<-POPL[i,j]*R1
		Y1[i,j]~dpois(mu1[i,j])
		log(mu1[i,j])<-log(exp1[i,j])+log(theta1[i,j])
		log(theta1[i,j])<-a01+p1[i]*modS1[i]+(1-p1[i])*modST1[i,j]
		ppoc1[i,j] <- exp(-mu1[i,j] + Y1[i,j]*log(mu1[i,j]) -
			logfact(Y1[i,j]) )
		# inverse of the conditional predictive ordinate
		icpoc1[i,j] <- 1/ppoc1[i,j]
		logppoc1[i,j]<-log(ppoc1[i,j])
		ll1[i,j]<-Y1[i,j]*log(mu1[i,j])-logfact(Y1[i,j])-mu1[i,j]
		exp2[i,j]<-POPL[i,j]*R2
		Y2[i,j]~dpois(mu2[i,j])
		log(mu2[i,j])<-log(exp2[i,j])+log(theta2[i,j])
```

```
		log(theta2[i,j])<-a02+p2[i]*modS2[i]+(1-p2[i])*modST2[i,j]
		ppoc2[i,j] <- exp(-mu2[i,j] + Y2[i,j]*log(mu2[i,j]) -
			logfact(Y2[i,j]) )
		# inverse of the conditional predictive ordinate
		icpoc2[i,j] <- 1/ppoc2[i,j]
		logppoc2[i,j]<-log(ppoc2[i,j])
		ll2[i,j]<-Y2[i,j]*log(mu2[i,j])-logfact(Y2[i,j])-mu2[i,j]
		exp3[i,j]<-POPL[i,j]*R3
		Y3[i,j]~dpois(mu3[i,j])
		log(mu3[i,j])<-log(exp3[i,j])+log(theta3[i,j])
		log(theta3[i,j])<-a03+p3[i]*modS3[i]+(1-p3[i])*modST3[i,j]
		ppoc3[i,j] <- exp(-mu3[i,j] + Y3[i,j]*log(mu3[i,j]) -
			logfact(Y3[i,j]) )
		# inverse of the conditional predictive ordinate
		icpoc3[i,j] <- 1/ppoc3[i,j]
		logppoc3[i,j]<-log(ppoc3[i,j])
		ll3[i,j]<-Y3[i,j]*log(mu3[i,j])-logfact(Y3[i,j])-mu3[i,j]
		modST1[i,j]<-pow(gam[j]*step(gam[j]),rho[1])+
			pow(gam[j]*(step(gam[j])-1),rho[1])*(-1)+
			phi1[i,j]+aST1[1,j]*xST1[i,j]+aST1[2,j]*xST2[i,j]+
			aST1[3,j]*xST3[i,j]+aT1*xT[j]
		modST2[i,j]<-pow(gam[j]*step(gam[j]),rho[2])+
			pow(gam[j]*(step(gam[j])-1),rho[2])*(-1)+
			phi2[i,j]+aST2[1,j]*xST1[i,j]+aST2[2,j]*xST2[i,j]+
			aST2[3,j]*xST3[i,j]+aT2*xT[j]
		modST3[i,j]<-pow(gam[j]*step(gam[j]),rho[3])+
			pow(gam[j]*(step(gam[j])-1),rho[3])*(-1)+
			phi3[i,j]+aST3[1,j]*xST1[i,j]+aST3[2,j]*xST2[i,j]+
			aST3[3,j]*xST3[i,j]+aT3*xT[j]
		phi1[i,j]~dnorm(0,tauphi1[j])
		phi2[i,j]~dnorm(0,tauphi2[j])
		phi3[i,j]~dnorm(0,tauphi3[j])
	}#close j loop
	modS1[i]<-v[i]+u1[i]+aS1[1]*xS1[i]+aS1[2]*xS2[i]+aS1[3]*xS3[i]
	modS2[i]<-v[i]+u2[i]+aS2[1]*xS1[i]+aS2[2]*xS2[i]+aS2[3]*xS3[i]
	modS3[i]<-v[i]+u3[i]+aS3[1]*xS1[i]+aS3[2]*xS2[i]+aS3[3]*xS3[i]
	u1[i]~dnorm(0,tauu1)
	u2[i]~dnorm(0,tauu2)
	u3[i]~dnorm(0,tauu3)
	logit(p1[i])<-z1[i]+alp1[i]
	alp1[i]~dnorm(0,taualp1)
	logit(p2[i])<-z2[i]+alp2[i]
```

```
		alp2[i]~dnorm(0,taualp2)
		logit(p3[i])<-z3[i]+alp3[i]
		alp3[i]~dnorm(0,taualp3)
}close i loop
d1<--2*sum(ll1[,])
d2<--2*sum(ll2[,])
d3<--2*sum(ll3[,])
z1[1:46]~car.normal(adj[],weights[],num[],taup1)
z2[1:46]~car.normal(adj[],weights[],num[],taup2)
z3[1:46]~car.normal(adj[],weights[],num[],taup3)
v[1:46]~car.normal(adj[],weights[],num[],tauv)
for(k in 1:sumNumNeigh){weights[k]<-1}
for(j in 1:14){
		tauphi1[j]<-pow(sdphi1[j],-2)
		tauphi2[j]<-pow(sdphi2[j],-2)
		tauphi3[j]<-pow(sdphi3[j],-2)
		sdphi1[j]~dunif(0,4)
		sdphi2[j]~dunif(0,4)
		sdphi3[j]~dunif(0,4)
		MPLc1[j]<-sum(logppoc1[,j])
		MPLc2[j]<-sum(logppoc2[,j])
		MPLc3[j]<-sum(logppoc3[,j])
}#close j loop
gam[1]~dnorm(0,taugam)
for (j in 2:14){
		gam[j]~dnorm(gam[j-1],taugam)
		}#close j loop
taup1<-pow(sdp1,-2)
sdp1~dunif(0,4)
taup2<-pow(sdp2,-2)
sdp2~dunif(0,4)
taup3<-pow(sdp3,-2)
sdp3~dunif(0,4)
tauv<-pow(sdv,-2)
sdv~dunif(0,4)
taugam<-pow(sdgam,-2)
sdgam~dunif(0,4)
a01~dnorm(0,tau01)
tau01<-pow(sd01,-2)
sd01~dunif(0,4)
a02~dnorm(0,tau02)
tau02<-pow(sd02,-2)
```

```
sd02~dunif(0,4)
a03~dnorm(0,tau03)
tau03<-pow(sd03,-2)
sd03~dunif(0,4)
tauu1<-pow(sdu1,-2)
sdu1~dunif(0,4)
tauu2<-pow(sdu2,-2)
sdu2~dunif(0,4)
tauu3<-pow(sdu3,-2)
sdu3~dunif(0,4)
taualp1<-pow(sdalp1,-2)
sdalp1~dunif(0,4)
taualp2<-pow(sdalp2,-2)
sdalp2~dunif(0,4)
taualp3<-pow(sdalp3,-2)
sdalp3~dunif(0,4)
aT1~dnorm(0,tauaT1)
tauaT1<-pow(sdaT1,-2)
sdaT1~dunif(0,4)
aT2~dnorm(0,tauaT2)
tauaT2<-pow(sdaT2,-2)
sdaT2~dunif(0,4)
aT3~dnorm(0,tauaT3)
tauaT3<-pow(sdaT3,-2)
sdaT3~dunif(0,4)
for (k in 1:3){
	aS1[k]~dnorm(0,tauaS1[k])
	tauaS1[k]<-pow(sdaS1[k],-2)
	sdaS1[k]~dunif(0,4)
	aS2[k]~dnorm(0,tauaS2[k])
	tauaS2[k]<-pow(sdaS2[k],-2)
	sdaS2[k]~dunif(0,4)
	aS3[k]~dnorm(0,tauaS3[k])
	tauaS3[k]<-pow(sdaS3[k],-2)
	sdaS3[k]~dunif(0,4)
	rho[k]~dgamma(2,1)
	for (j in 1:14){
		aST1[k,j]~dnorm(0,tauaST1[k])
		aST2[k,j]~dnorm(0,tauaST2[k])
		aST3[k,j]~dnorm(0,tauaST3[k])
	}#close j loop
		tauaST1[k]<-pow(sdaST1[k],-2)
```

```
                sdaST1[k]~dunif(0,4)
                tauaST2[k]<-pow(sdaST2[k],-2)
                sdaST2[k]~dunif(0,4)
                tauaST3[k]<-pow(sdaST3[k],-2)
                sdaST3[k]~dunif(0,4)
}#cloose k loop
}#close model loop
```
